# Recent Progress on Vanadium Dioxide Nanostructures and Devices: Fabrication, Properties, Applications and Perspectives

**DOI:** 10.3390/nano11020338

**Published:** 2021-01-28

**Authors:** Yanqing Zhang, Weiming Xiong, Weijin Chen, Yue Zheng

**Affiliations:** 1 State Key Laboratory of Optoelectronic Materials and Technologies, School of Physics, Sun Yat-sen University, Guangzhou 510275, China; zhangyq66@mail2.sysu.edu.cn (Y.Z.); chenweijin@mail.sysu.edu.cn (W.C.); 2 Centre for Physical Mechanics and Biophysics, School of Physics, Sun Yat-sen University, Guangzhou 510275, China; 3 School of Materials, Sun Yat-sen University, Guangzhou 510275, China

**Keywords:** vanadium dioxide, nanostructures, metal-insulator phase transition, fabrication methods, properties and related applications

## Abstract

Vanadium dioxide (VO_2_) is a typical metal-insulator transition (MIT) material, which changes from room-temperature monoclinic insulating phase to high-temperature rutile metallic phase. The phase transition of VO_2_ is accompanied by sudden changes in conductance and optical transmittance. Due to the excellent phase transition characteristics of VO_2_, it has been widely studied in the applications of electric and optical devices, smart windows, sensors, actuators, etc. In this review, we provide a summary about several phases of VO_2_ and their corresponding structural features, the typical fabrication methods of VO_2_ nanostructures (e.g., thin film and low-dimensional structures (LDSs)) and the properties and related applications of VO_2_. In addition, the challenges and opportunities for VO_2_ in future studies and applications are also discussed.

## 1. Introduction

A phase transition is a sudden change of one phase to another under an external stimuli, e.g., a thermal field, strain energy, surface energy, an external force, a magnetic field, etc., accompanied by the significant change in physical properties. Metal–insulator transition (MIT) is one of the typical phase transition types in oxides, and has been a research hotspot in recent decades. Among inorganic materials with MIT, vanadium dioxide (VO_2_) is widely focused on because of the near-room-temperature phase transition temperature (*T_c_* ≈ 340 K) and the reversible, huge changes in conductance and transmittance during MIT [1,2]. This reversible phase transition is a first-order phase transition, which is accompanied by a crystal structure change from a low-temperature monoclinic phase to a high-temperature rutile phase. Besides, the strongly correlated electron effect introduced by the special *d* electron orbit structure of VO_2_ leads to abundant interesting physical and chemical properties. Based on these excellent performances, VO_2_ can be widely used in many fields and has been becoming one of the hottest metal oxide materials in recent years [3,4,5,6,7,8,9]. Moreover, the rapid development in VO_2_ preparation and performance modulation technologies has greatly promoted the application of VO_2_ in many aspects; e.g., VO_2_ can be used as a channel layer of field-effect transistor (FET) due to its electric-field-adjustable MIT behavior. The distinctive hysteresis loop during the MIT process under an electric filed or temperature change leads to applications in memory devices; the great difference of transmittance between the insulating and metallic phases makes it a candidate material for smart windows; the strain and gas-environment-dependent MIT behavior holds promise for developing novel strain and gas sensors, etc., [6,10,11,12,13].

With the development of material fabrication technology, various VO_2_ nanostructures, e.g., thin films and low-dimensional structures (LDSs), have been successfully fabricated and have been widely investigated. Thin films and LDSs are suitable for micromachining processes, which promotes the development of both macro- and micro-scale devices. As we know, the MIT temperature and behavior of VO_2_ are extremely sensitive to external stimuli, including doping, strain, surface/interface effects, electrochemical gating, electric field, light, electron beam, etc. [6,14,15,16,17,18,19,20]. Besides, the morphology also has a great influence on the properties of VO_2_ material. VO_2_ LDSs—nanowires (NWs), nanodots (NDs), nanoparticles (NPs), nanorods (NRs), nanobelts (NBs), nanosheets (NSs), etc.—exhibit unique electrical, mechanical and optical properties different from their bulk counterparts due to the size and surface/interface effects. Recently, VO_2_ thin films and LDSs have been successfully fabricated via advanced growth techniques and have attracted much attention.

On the basis of a survey of excellent experimental works on VO_2_, we aim to provide a wide range of insights into the recent studies in this field. In Section 2, the multiple phases of VO_2_ are primarily introduced. We emphasize the performances of different phases and the transform paths among them. In Section 3, several commonly used methods—e.g., the hydrothermal method, chemical vapor deposition (CVD), pulsed laser deposition (PLD), sol–gel, magnetron sputtering, electrospinning and molecular beam epitaxy (MBE) are introduced. These methods have unique characteristics in the fabrication of VO_2_ nanostructures and can be employed to realize abound VO_2_ nanostructures, including NDs, NWs, NRs, NSs, NPs, nanoplates, nanorings, thin films, etc. In Section 4, the properties and the applications of VO_2_ nanostructures are summarized and discussed. Finally, the challenges and opportunities of VO_2_ in future studies and applications are also discussed.

## 2. Overview of VO_2_ Polymorphs

**Figure 1:**
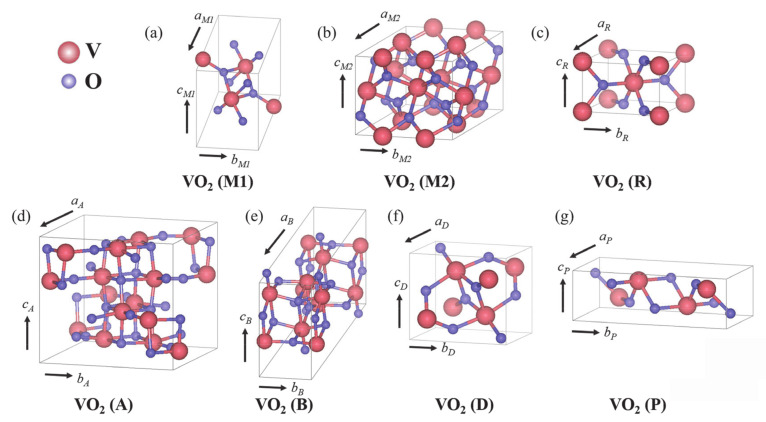
The crystal structures of VO_2_ polymorphs, (**a**) VO_2_ (M1), (**b**) VO_2_ (M2), (**c**) VO_2_ (R), (**d**) VO_2_ (A), (**e**) VO_2_ (B), (**f**) VO_2_ (P) and (**g**) VO_2_ (D). The corresponding ICSD collection codes of crystal structures are 34033 [VO_2_ (M1)], 34417 [VO_2_ (M2)], 4110 [VO_2_ (R)], 57155 [VO_2_ (A)], 73855 [VO_2_ (B)] and 22303 [VO_2_ (P)], and the crystal structure of VO_2_ (D) is modified from [25], with permission from Royal Society of Chemistry, 2012.

**Figure 2:**
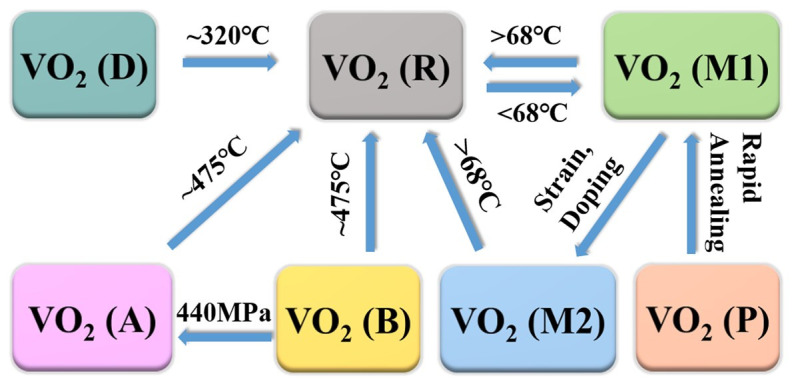
Diagram of phase transformations between different phases of VO_2_ [25,26,31,32,33].

At present, several phases of VO_2_ have been reported, including VO_2_ (M) (*P* 2_1_/*c*), VO_2_ (R) (*P* 4_2_/*mnm*), VO_2_ (A) (*P* 4_2_/*nmc*), VO_2_ (B) (*C* 2/*m*), VO_2_ (C) (*I* 4/*mmm*), VO_2_ (D) (*P* 2/c) and VO_2_ (P) (*Pbnm*), as shown in Table 1 [21,22,23,24,25,26,27,28,29,30]. Additionally, the corresponding crystal structures are displayed in Figure 1. Under certain conditions, these phases can be transformed into each other (Figure 2) [25,26,31,32,33].

Among these phases, VO_2_ (M), VO_2_ (R), VO_2_ (A) and VO_2_ (B) have the most common crystal structures. The basic structural unit is the VO6 octahedron—i.e., a vanadium atom is in the center of the octahedron and is surrounded by six oxygen atoms. Octahedrons are arranged by common edges or vertices, and a long-range ordered structure is formed in three-dimensional space. For VO_2_ crystals with different crystal structures, the shape of the VO6 octahedron and the connection modes between octahedrons are different, which leads to the differences of atomic coordinates and spatial symmetry groups. VO_2_ (M) is an insulating monoclinic phase containing VO_2_ (M1) and VO_2_ (M2) phases. Generally, the MIT of VO_2_ is the transition of VO_2_ (M1) into VO_2_ (R) after exceeding the MIT temperature. The low-temperature monoclinic phase is an insulating phase (M or M1 phase, space group *P* 2_1_/*c*, *a*_M_ = 5.715 Å, *b*_M_ = 4.554 Å, *c*_M_ = 5.385 Å, β = 122.6°) and the high-temperature rutile phase is a metal phase (R phase, space group *P* 4_2_/*mnm*, *a*_R_ = *b*_R_ = 4.554 Å, *c*_R_ = 2.85 Å, β = 90°) [21,22]. In the low-temperature monoclinic phase, V atoms form zigzag structure along the direction of *c* axis. In the high-temperature rutile phase, V atoms form a straight line along the direction of *c* axis with the period of 1/2 × *c*_M_. During the phase transition from insulating phase to metal phase, the octahedral structure composed of V–O changes from the partial octahedron at a low temperature to the normal octahedron at a high temperature, and the V–O bond angle changes from 90° to 78–99° (Figure 1c). In addition, compared with the high-temperature tetragonal phase, the lattice constants *a* and *b* of M phase are increased by ~0.6% and ~0.4%, respectively. Meanwhile, *c* is decreased by ~1.0%, and the change of volume is about −0.044%. Such a change in structure inevitably leads to the change of their macroscopic properties, e.g., electrical, optical, magnetic and mechanical properties. VO_2_ (M1) and VO_2_ (M2) both are insulating phases with monoclinic structures, and the difference between these two phases is that V atoms in VO_2_ (M1) form zigzag chain V–V pairs along the *c* axis. V atoms in VO_2_ (M2) have two forms along the *c* axis: one is that V atom chains are paired but not inclined (relative to the *c* axis); the other is that V atom chains are inclined but not paired (Figure 1a,b). VO_2_ (M1) can be transformed into VO_2_ (M2) by applying strain along (1–10) direction or doping with +3 valence ions [31]. In addition, VO_2_ (M2) is usually regarded as an intermediate phase of VO_2_ (M1) (mainly in NWs) phase transition to VO_2_ (R) [34].

For VO_2_ (A), four sets of two-sided octahedral pairs on the *c* plane of VO_2_ (A) form a 2 × 2 square. Blocks of 2 × 2 along the *c* axis are stacked in the form of common edges to form Z-shaped long chains of V atoms (Figure 1d). Except for VO_2_ (M), VO_2_ (A) also has been proven to have obvious MIT behavior. VO_2_ (A) has a typical first-order phase transition with *T_c_* near 435 K, which is higher than VO_2_ (M). During the phase transition in VO_2_ (A), the resistance has a sudden change of about 1–2 orders of magnitude [29]. On the other hand, unlike monoclinic VO_2_ (M) and VO_2_ (A), VO_2_ (B) has no obvious MIT characteristic or abrupt changes in optical and electric properties. The *b*-axis direction of VO_2_ (B) has two octahedrons; the second layer moves 1/2, 1/2 and 0 (fractional coordinates in unit cell) relative to the first layer; and octahedrons are linked by common edges or vertices (Figure 1e). VO_2_ (B) has excellent temperature coefficient resistance (TCR) of −7%/K near room temperature and a suitable square sheet resistance of 20–50 KΩ [35]. Therefore, VO_2_ (B) is suitable for using as an electrode material and a thermal sensitive material for batteries. Under the pressure of 440 MPa, VO_2_ (B) could be transformed into VO_2_ (A) [32]. Moreover, VO_2_ (A) and VO_2_ (B) can be transformed into VO_2_ (R) after annealing at 475 °C for 1 h under the protection of argon (Ar) gas [33].

VO_2_ (C) was found by Hagrman et al. and synthesized through the hydrothermal method [28]. The structure of VO_2_ (C) consists of VO_5_ square pyramids, each of which shares its four base edges with four adjacent VO_5_ square pyramids. However, there are few studies on VO_2_ (C), and more studies are needed to reveal its physical and chemical properties. In addition, Liu et al. [25] synthesized a micro/nanostructure consisting of NSs with a new phase of VO_2_ (D). They demonstrated that the formation energy of VO_2_ (D) was close to rutile-type VO_2_ (R), and VO_2_ (D) can be transformed into VO_2_ (R) by annealing at 320 °C for 2 h in the protection gas. After that, VO_2_ (M) can be obtained after a cooling process. Moreover, the VO_2_ (D) exhibits Arrhenius-type behavior with a bandgap of 0.33 eV. Temperature-dependent magnetic susceptibility measurements demonstrated the magnetic properties of VO_2_ (D). This provides potential applications of VO_2_ in magnetic and electronic devices. Besides, there is another new phase of VO_2_ (P) that was synthesized by a simple chemical reaction route [26]. Additionally, it was demonstrated that VO_2_ (P) can be transformed into VO_2_ (M) by a rapid annealing process.

## 3. Methods for the Growth of VO_2_ Nanostructures

Driven by the advanced instruments and techniques for the growth of materials, the growth of VO_2_ has made great progress during the past decades. As a transition metal element, vanadium contains several valence states, e.g., V^2+^, V^4+^ and V^5+^ [3,16,36]. The vanadium element in VO_2_ is +4 valence; thus, it is easy to be oxidized in the process of preparation and to get mixed VO_2_/V_2_O_5_ products with +4 and +5 valences. In recent years, various techniques have been carried out for the growth of VO_2_ with a pure phase, such as sputtering, PLD, sol–gel method, CVD, hydrothermal method and electrospinning [33,37,38,39,40,41,42]. Each method has its features and suits the growth of different structures. In this section, growth methods of VO_2_ will be systematically illustrated and discussed.

### 3.1. Hydrothermal Method

The hydrothermal method can be dated back to 1845, which was established for simulating nature mineralization. In the hydrothermal method, oxides, hydrides and gels are usually used as the source materials. Under the high temperature and high-pressure environment, the source materials are dissolved in the solvent and chemical reactions will occur. The products are oversaturated and then crystallized out to form various LDSs with different morphologies. Owing to surface and interface effects, LDSs exhibit novel physical and chemical properties, which are widely investigated in scientific studies and industrial engineering. Due to the unique advantages in LDS synthetization, the hydrothermal method has been widely used. For the numerous materials, the hydrothermal method can effectively synthesize LDSs with various morphologies. The morphologies and crystalline phases of hydrothermal products are affected by various factors, e.g., precursors, temperature, pH value, filling degree and doping [43,44,45,46]. Besides, the addition of surfactants in the reaction process, such as polyethylene glycol (PEG) and polyvinylpyrrolidone (PVP), can effectively improve the crystallinity of products and promote the growth of specific morphologies [29]. The hydrothermal method has many advantages, e.g., good crystallinity, wide application range, simple experimental process and high yield. However, it also has some shortcomings—poor reproducibility, narrow reaction temperature, high risk (high pressure) and so on.

The hydrothermal method is an efficient and effective approach for synthesizing LDSs of VO_2_. Presently, the hydrothermal method has been successfully used to synthesize various VO_2_ LDSs, e.g., NPs, NWs, NRs, NSs, nanoflowers, etc. Generally, for the hydrothermal synthetization of VO_2_ LDSs, pentavalent vanadium is used as the vanadium source—V_2_O_5_, ammonium metavanadate, etc.—and alcohols are used as reducing agents. VO_2_ LDSs with different morphologies and sizes can be obtained by controlling the parameters during the process of synthetization, e.g., reaction temperature, time and the type of reducing agent.

#### 3.1.1. The Growth of VO_2_ Nanoparticles (NPs)

When undergoing a reversible MIT, VO_2_ has remarkable changes in infrared transmittance, which makes it attract much attention in the applications of the energy-saving field (e.g., smart windows) [47]. For the thermochromic smart windows based on VO_2_ (M), the luminous transmittance and solar energy modification ability are the two main parameters [11]. Theoretical calculation results showed that better luminous transmittance and solar energy transmittance modulation could be achieved by reducing the size of VO_2_ (M) NPs to smaller than the wavelength of visible light [48]. Besides, among all nanostructures, sub-100 nm 0-dimensional (0D) VO_2_ (M) NPs have been studied and showed excellent monodispersity and thermochromic performance.

Over the past decade, researchers have extensively studied the preparation of 0D VO_2_ (M) nanostructures by the hydrothermal method. However, the polymorphism of VO_2_ makes it hard to synthesize pure phase VO_2_ (M) in one step. Thus, a subsequent thermal treatment is necessary to form pure phase VO_2_ (M). It has been reported that VO_2_ (M) NPs with an average particle size less than 100 nm can be prepared by the hydrothermal method. Li et al. [49] firstly synthesized VO_2_ (D) NPs by using oxalic acid dehydrate as the reducing agent and polyvinylalcohol as the surfactant. Then, the VO_2_ (M) NPs were obtained by annealing in a vacuum environment (~20 Pa) at different temperatures for 1 h. The as-prepared VO_2_ (M) NPs were uniformly distributed with the average size of 70 nm.

In order to better control the size of VO_2_ NPs, Chen et al. [50] optimized the preparation process of VO_2_ NPs and synthesized high-quality VO_2_ NPs with smaller diameters (i.e., 25–40 nm). In this work, vanadium pentoxide and diamide hydrochloride were used as source materials. Here, the synthesis of VO_2_ NPs is considered to be a “heating-up” process. When the precursor was heated to the critical temperature, the precursor was instantaneously decomposed to produce excessive monomers, a highly supersaturated reaction system was formed and then a burst-nucleation process occurred. In this process, a large number of nuclei simultaneously grew and rapidly consumed monomers, which inhibited the further growth of particles.

**Figure 3:**
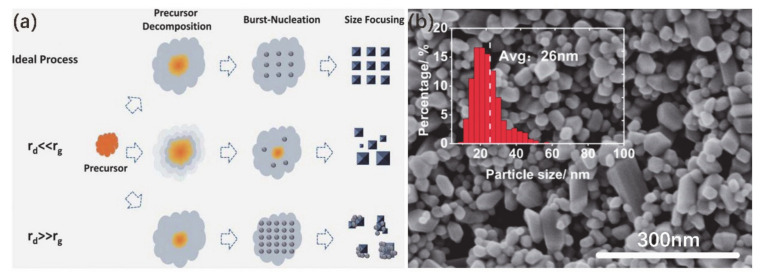
(**a**) The schematic illustration of growth of VO_2_ nanoparticles (NPs) under the ideal “heating-up” process and under the situations of r_d_ ≪ r_g_ and r_d_ ≫ r_g_. (**b**) SEM image of grown VO_2_ (M) NPs; the inset in (**b**) shows the size distribution profile from more than 300 particles. Reproduced from [50], with the permission from Royal Society of Chemistry, 2014.

During the process of growth, the decomposition rate of the precursor (*r*_d_) and the growth rate of the grain (*r*_g_) are two important factors that determine the particle size, as shown in Figure 3a. When *r*_d_ ≪ *r*_g_, the growth of smaller grains is restricted because of the absence of monomers, which results in the polarization of grain size. If *r*_d_ ≫ *r*_g_, the supersaturated monomer solution produces excessive nuclei and leads to aggregation as the concentration increases. Thus, in order to precisely control the size of NPs, it is a key point to control the *r*_d_ and *r*_g_ in the growth process. Finally, the hydrothermal products were annealed at a higher temperature to obtain VO_2_ (M). Figure 3b shows the scanning electron microscope (SEM) image of VO_2_ (M) NPs with good crystallinity and a relatively low average size of 23 nm. This method can provide a reference for the growth of VO_2_ NPs with ultra-small size.

#### 3.1.2. The Growth of VO_2_ Nanowires/Nanorods (NWs/NRs)

**Figure 4:**
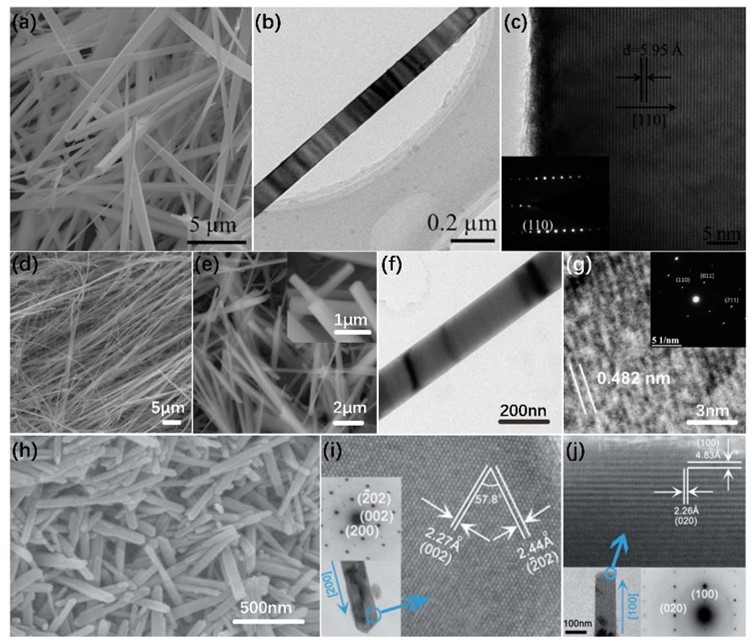
(**a**) SEM image of the VO_2_ (A) nanowires (NWs) and (**b**) low-resolution TEM image of individual VO_2_ (A) NW. (**c**) HRTEM image of the end of the VO_2_ (A) NW, and the inset is the corresponding SAED pattern. Reproduced from [29], with permission from Royal Society of Chemistry, 2014. (**d**) SEM image of V_3_O_7_·H_2_O NWs. (**e**) SEM image of monoclinic VO_2_ (M) NWs; the inset in (**e**) indicates rectangular cross-sections of VO_2_ (M) NWs. (**f**) TEM image of a monoclinic VO_2_ (M) NW. (**g**) HRTEM image of a monoclinic M1 phase VO_2_ NW; the inset in (**d**) shows the corresponding SAED pattern. Reproduced from [33], with permission from American Chemical Society, 2014. (**h**) SEM image of VO_2_ (M) NRs. (**i**,**j**) HRTEM images and SAED patterns of two individual VO_2_ (M) nanorods (NRs); insets are their corresponding SAED patterns and the morphologies of the selected NRs. Reproduced from [54], with permission from Royal Society of Chemistry, 2011.

The synthesis and characterization of one-dimensional (1D) nanostructures, e.g., NWs and NRs, have attracted much attention due to their anisotropic surface properties and potential applications in integrated devices. Moreover, 1D nanostructures can offer large specific surface areas (defined as the surface area per unit volume) and efficient electron transport pathways to achieve high capacity. Thus, 1D VO_2_ has been extensively studied in recent years. Several studies have reported the preparation of 1D VO_2_, especially NWs, NRs, etc., via the hydrothermal method [33,51,52,53,54,55]. The most important feature of the hydrothermal method is that the free-standing VO_2_ nanostructures can be grown without substrates, which facilitates the dispersal of the NWs in further applications. It is worth noting that most of the VO_2_ structures synthesized by hydrothermal method are VO_2_ (A) or VO_2_ (B) [29,30,52,53,54,56]. In our previous work, single crystallized VO_2_ (A) NWs were synthesized by V_2_O_5_; the oxalic acid (H_2_C_2_O_2_·H_2_O) and PEG-6000 were used as the reducing agent and surfactant, respectively [29]. The morphology of VO_2_ (A) characterized by SEM is shown in Figure 4a. The width of NWs ranges from tens to hundreds of nanometers, and length is from a few microns to tens of microns. Figure 4b,c displays low-resolution transmission electron microscopy (LRTEM) and high-resolution (HRTEM) images of an individual VO_2_(A) NW. According to the figures, the spacing between the lattice fringes of 5.95 Å exactly corresponds to the distance between two (110) crystal planes of VO_2_ (A). With the moving of the electron beam along the NW, the selected area electron diffraction (SAED) pattern (inset of Figure 4b) remains unchanged, indicating the whole NW is single crystal. In order to form VO_2_ (M), the further annealing process is needed. Horrocks et al. [33] studied the synthesis of free-standing VO_2_ (M) via hydrothermal method in three steps. Firstly, V_3_O_7_·H_2_O NWs were synthesized through the hydrothermal exfoliation and reduction of bulk V_2_O_5_ by using oxalic acid dihydrate, as shown in Figure 4d. The NWs were 183 ± 34 nm in width and hundreds of micrometers in length. Then, V_3_O_7_·H_2_O NWs were hydrothermally reduced to form VO_2_ (A) and VO_2_ (B) NWs. Finally, the NWs mixtures of VO_2_ (A) and VO_2_ (B) were collected and then annealed at 475 °C for 1 h to crystallize into M1 phase. Figure 4e shows the SEM image of annealed monoclinic VO_2_ (M) NWs with the rectangular cross-section and the length of about tens of microns. The TEM image (Figure 4f) of the NWs shows the average width of 187 ± 77 nm, which is close to the width of V_3_O_7_·H_2_O NWs. This indicates that the morphology of VO_2_ (M) NWs could not be affected by the annealing process. The HRTEM image and SAED pattern of single NW confirm that the M1 monoclinic phase exists in VO_2_ NW, as shown in Figure 4g. Besides. Wu et al. [54] reported a controllable oxidation reaction to synthesize the highly uniform VO_2_ (M) NRs. Firstly, they synthesized the V(OH)_2_NH_2_ precursor by a controllable oxidation reaction of V(OH)_2_NH_2_ in formic acid (HCOOH) buffer solution [57]. Additionally, the HNO_3_ solution was added to the V(OH)_2_NH_2_ precursor and stirred strongly. After that, the mixture was heated at the temperature of 200 °C for 36 h. Finally, after cooling the system to room temperature, the final VO_2_ (M) sample was collected by centrifugation. Figure 4h shows the SEM image of as-grown VO_2_ (M) NRs. The diameter of NRs ranged from 30 to 120 nm, and the length was up to 400–800 nm. Figure 4i,j shows the HRTEM images of two individual VO_2_ (M) NRs. Insets are their corresponding SAED patterns and the morphologies of the selected NRs. The results clearly reveal the crystallographic growth model for monoclinic VO_2_ (M) NRs.

#### 3.1.3. The Growth of VO_2_ Nanosheets (NSs)

**Figure 5:**
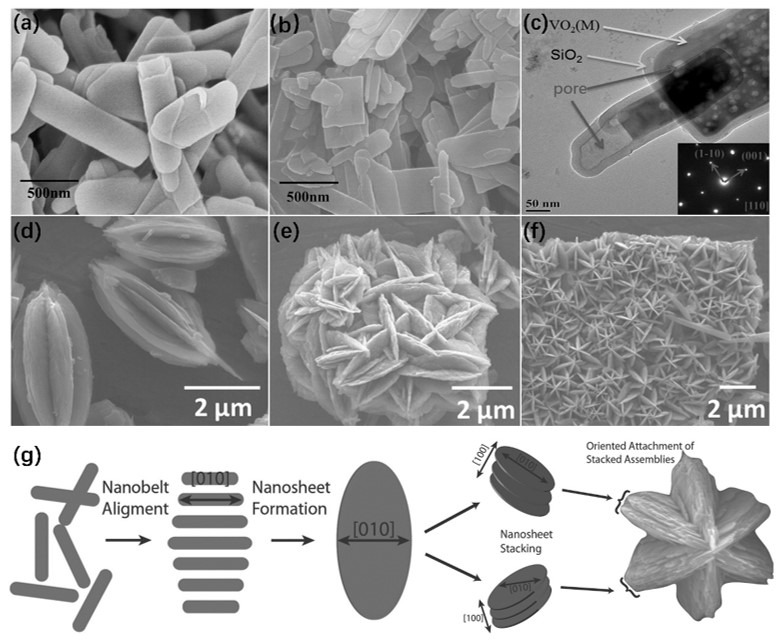
(**a**) SEM image of VO_2_ (B)@SiO_2_ NPs. (**b**) SEM image of VO_2_ (M) annealing from VO_2_ (B). (**c**) TEM image of VO_2_ (M)@SiO_2_; the inset in (**c**) is the corresponding SAED pattern. Reproduced from [58], with permission from Elsevier, 2013. (**d**–**f**) Representative individual mesocrystal assemblies and an overall SEM micrograph of a typical specimen. (**g**) Schematic diagram of the formation mechanism of VO_2_ (B) mesocrystals. Reproduced from [59], with permission from John Wiley and Sons, 2013.

The VO_2_ synthesized via hydrothermal method also easily forms NSs or “flower-like” structures [43,58,59]. Li et al. [58] fabricated plate-like VO_2_ (M)@SiO_2_ NSs via low-temperature hydrothermal method with post-annealing. The VO_2_ (B) coated with SiO_2_ was firstly obtained by using the modified Stober method. The length or width of VO_2_ (B)@SiO_2_ NSs was several hundreds of nanometers, and the thickness was 20–30 nm, as shown in Figure 5a. Moreover, the VO_2_ (M)@SiO_2_ NSs can be obtained from VO_2_ (B)@SiO_2_ by a heat treatment process, and the morphologies of the products are very close to the original morphology of VO_2_ (B)@SiO_2_, as illustrated in Figure 5b. The TEM image of VO_2_ (M)@SiO_2_ in Figure 5c shows that a thin layer of SiO_2_ was uniformly coated on VO_2_ (M), and numerous nanopores were formed inside. The density difference between VO_2_ (M) and VO_2_ (B) leads to the formation of nanopores. In recent years, flower-like or star-assemblies structures consisting of NSs have been investigated [60,61]. These structures have larger specific surface areas and may be used in some specific fields. Uchaker et al. used the acid reduction approach to synthesize star-assemblies of VO_2_ (B) mesocrystals [59], which were used as a cathodic electrode material in lithium-ion batteries. Figure 5d–f illustrates the representative individual mesocrystal assemblies and the corresponding overall SEM micrograph. The star-assemblies were evenly distributed and closely arranged. Additionally, the size of VO_2_ (B) NSs was about tens of nanometers. Each arm of the star assembly was separated from the other ones, indicating that it was not a single crystal structure. The VO_2_ (B) nanostructures reported in this work were synthesized by the following reaction: V_2_O_5_ + H_2_C_2_O_4_ →VO_2_ (B) + H_2_O + CO_2_. Oxalic acid was employed as both the reducing agent and the chelating agent. A schematic diagram of the formation mechanism of the mesocrystal is shown in Figure 5g. Firstly, VO_2_ (B) is treated with the solvothermal method for 4 h to form elliptical NSs. The growth direction of VO_2_ (B) does not change during this step. Then, several NSs are stacked together in an epitaxial manner to minimize the system’s energy. The residual chelating agent remaining on the (100) surface of each NS can effectively reduce the Debye length of the electric double layer, and the van der Waals forces between the components can be stabilized, thereby leading to the formation of the superstructure and preventing the fusion of individual NSs. After this step, several VO_2_ (B) NSs are stacked together to form a star-like structure.

#### 3.1.4. The Growth of VO_2_ Nanorings

**Figure 6:**
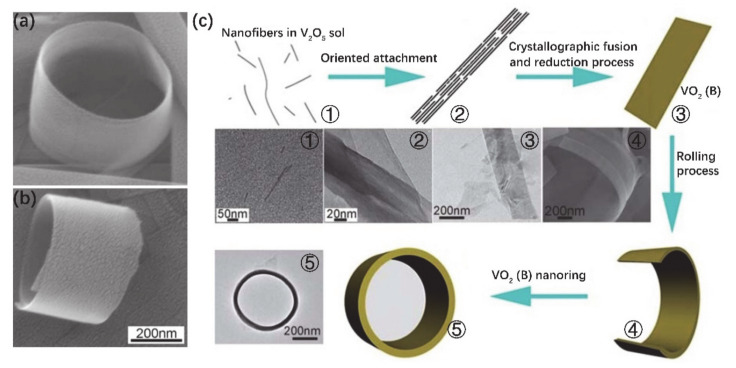
(**a**,**b**) SEM images of a single VO_2_ (B) nanoring viewed from different directions. (**c**) Schematic diagrams of the formation processes of VO_2_ (B) nanorings. Reproduced from [64], with permission from Royal Society of Chemistry, 2011.

In recent decades, it has still been a challenge to synthetize complex nanostructures, e.g., a nanoring structure. The special morphology of nanorings makes them exhibit many unusual behaviors, such as the abnormal dispersion of a magnetic field in semiconductor nanorings [62,63,64,65]. Li et al. [64] synthesized metastable phase VO_2_ (B) nanorings by hydrothermal treatment of V_2_O_5_ sol with PEG. PEG was employed as a surfactant and reducing agent, which is important for adjusting the length and width of VO_2_ NBs. It was found that proper hydrothermal conditions can accelerate the directional attachment growth of nanofibers in precursors and lead to the formation of VO_2_ (B) nanorings. SEM images in Figure 6a,b display different perspectives of a single VO_2_ (B) nanoring. The diameter and shell thickness of the as-grown VO_2_ (B) nanorings are 300–500 nm and 10–40 nm, respectively. The corresponding schematic diagram of formation processes is displayed in Figure 6c. VO_2_ (B) has a lamellar structure, and hydrothermal conditions provide a driving force for the formation of nanoring structures. In this work, PEG had a uniform and ordered chain structure (hydrophilic –O– and hydrophobic –CH_2_–CH_2_– radicals half-and-half), which is easy to adsorb on the surface of metal oxide colloids and reduces the colloid activities. With the increase of polymer absorption, the growth rate of colloids will be limited. On the other hand, the intercalation properties of V_2_O_5_ make the organic molecules easily embed in their layered structures. PEG has been employed as a surfactant and reducing agent that effectively depresses the dimension of the intermediate product. Generally, the morphology and size of as-grown vanadium oxide nanostructures depend on the growth conditions, e.g., concentration and type of template. In this work, the concentrations of V_2_O_5_ sol and PEG were modified to control the thickness and length of the VO_2_ (B) nanoribbons, which act as predecessors of the nanorings. Stage 1 in Figure 6c shows the fibrous structure in V_2_O_5_ sol. Under hydrothermal conditions, these tiny fibers can maintain and even enhance the spontaneous oriented growth of VO_2_ (B) nanoribbon. At the beginning, self-assembly of V_2_O_5_ nanofibers resulted in the formation of banded structures with limited size in the presence of excessive PEG (stage 2 in Figure 6c). With the increase of hydrothermal time, a thin single-crystal VO_2_ (B) nanoribbon was formed by the crystallographic melting process (stage 3 in Figure 6c). Finally, VO_2_ (B) nanorings were formed by the rolling mechanism of nanoribbons (stages 4 and 5 in Figure 6c).

#### 3.1.5. The Growth of Other Structures

**Figure 7:**
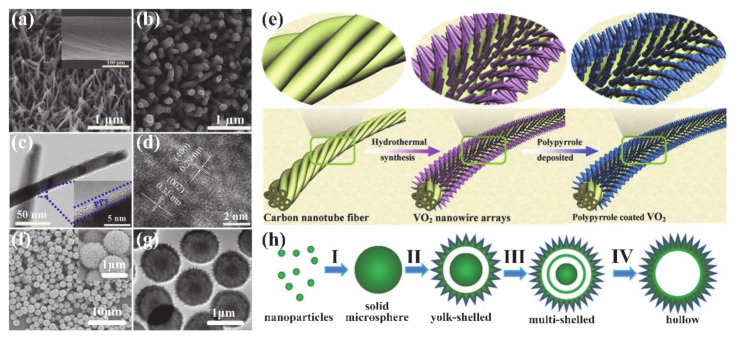
(**a**) SEM images of VO_2_ NWAs; the inset in (**a**) is a low-magnification SEM image of the VO_2_/CNTF hybrid fibers. (**b**) SEM image of VO_2_@PPy NWAs. (**c**) Low-magnification TEM image of VO_2_@PPy. (**d**) High-resolution TEM image of VO_2_. (**e**) Schematic diagram of the fabrication process about the VO_2_@PPy/CNTF electrode. Reproduced from [66], with permission from Elsevier, 2018. (**f**) Low-magnification SEM image of VO_2_; inset is a high-magnification SEM image. (**g**) Corresponding TEM image. (**h**) Structural evolution of the VO_2_ microspheres. Reproduced from [67], with permission from John Wiley and Sons, 2013.

In addition to the structures described above, some special structures, e.g., NW arrays (NWAs) and hollow microspheres, have been successfully fabricated by the hydrothermal method [66,67]. Man et al. [66] fabricated a fiber-shaped asymmetric supercapacitor (FASC) by growing aligned three-dimensional (3D) VO_2_@polypyrrole (VO_2_@PPy) core-shell NWAs on carbon nanotube fibers (CNTFs). Precursor solution and CNTF were placed together in an autoclave. The autoclave was then sealed and heated to 180 °C for 24 h. After the reaction was completed and the system was cooled to room temperature, the CNTFs covered with VO_2_ NWAs were removed and dried at 60 °C for 12 h. Finally, the VO_2_@PPy/CNTF cathode and a thin layer of nitrogen doped carbon coated vanadium nitride NWAs on CNTF anode were twisted together to form the all-solid-state FASC devices after drying. The schematic diagram of the fabrication process of the VO_2_@PPy/CNTF electrode is displayed in Figure 7e. Figure 7a shows that VO_2_ NWAs are aligned and densely distributed on the whole surface of the CNTF (inset of Figure 7a). Additionally, Figure 7b shows a SEM image of VO_2_@PPy NWAs. The TEM image in Figure 7c indicates that the VO_2_@PPy is a core-shell structure. VO_2_ NWA is continuously coated by a PPy thin shell with a thickness of 2–3 nm. In addition, HRTEM image in Figure 7d shows that the fringe spacings are about 0.29 and 0.31 nm, which correspond to the (400) and (002) planes of monoclinic VO_2_, respectively.

In recent years, hollow micro-nanostructures have attracted great interest due to their wide applications in many fields [68,69,70]. A great deal of work has reported the synthesis of hollow structures with different internal structures, which are attractive for many applications, e.g., drug delivery [71], photocatalysis [72], dye-sensitized solar cells [73], gas sensors [74] and lithium-ion batteries [67]. Pan et al. [67] synthesized various uniform VO_2_ microspheres with different hollow structures, e.g., yolk-shell, multi-shell and single-shell structures, via hydrothermal method. Figure 7f,g shows the SEM and TEM images of as-grown VO_2_ microspheres. The surface of a microsphere consists of small nanoplates with a thickness of about 20 nm. VO_2_ microspheres have a yolk-shell structure, and the shell thickness and spherical core diameter are 100 and 400 nm, respectively. Figure 7h shows the growth route of the VO_2_ microspheres and the corresponding structural evolution. Firstly, VOC_2_O_4_ was hydrolyzed as vanadium oxide NPs, and then they were fused to form solid microspheres in stage I. After that, the solid spheres underwent the inside-out Ostwald-ripening process for the first time and transformed into yolk-shells in stage II. With increase the solvothermal reaction time, the pre-formed solid cores underwent the secondary Ostwald-ripening process and transformed into a multi-shelled structure (stage III). Finally, the unstable internal structure was completely dissolved and recrystallized to form the hollow microspheres.

### 3.2. Chemical Vapor Deposition (CVD)

CVD is a chemical vapor reaction growth method that is one of the most commonly used deposition technologies. The CVD method can be traced back to 1945 when it was used to deposit TiC hard coatings. Generally, during the process of the chemical vapor reaction, a variety of reactants are gasified at high temperatures and then are carried by carrier gases to the surface of the substrate in the reaction chamber. Finally, the products are deposited on the surface of the substrate and grow into a thin film or other nanostructures. The deposition parameters of CVD, i.e., the types of gases, gas flow, reaction time, temperature and heating–cooling curve, have significant effects on the morphology and quality of the products. Among them, temperature is the most important factor. Too high a deposition temperature would cause the phenomenon of coarse grains. On the contrary, too low a temperature would cause an incomplete reaction, resulting in unstable structures and intermediate products, and a decrease of the bonding strength between products and substrates. Therefore, it is very important to set reasonable parameters during the growth process of materials. Various materials, including oxides, nitrides, carbides and elemental substances, can be deposited by CVD. In recent years, the growth of VO_2_ films and NWs by CVD method has been established.

#### 3.2.1. The Growth of VO_2_ Thin Films

**Figure 8:**
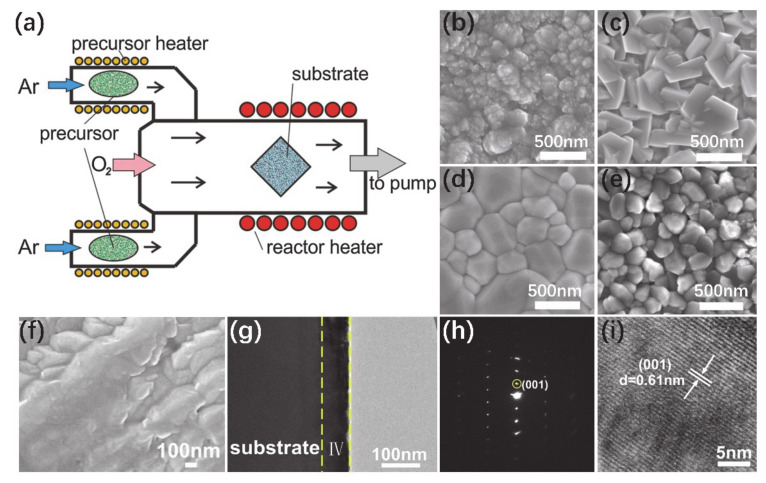
(**a**) Scheme of the CVD method for VO_2_ film growth. (**b**–**e**) SEM images of VO_2_ films deposited at various substrate temperatures. Reproduced from [75], with permission from Springer Nature, 2017. (**f**) SEM image of Mo-Al co-doped VO_2_ (B) thin films. (**g**–**i**) Cross-sectional TEM and SAED patterns and the corresponding HRTEM images. Reproduced from [76], with permission from Elsevier, 2018.

V(C_2_H_7_O)_4_, VOCl_3_, VCl_4_, VO(OC_3_H_7_)_7_ and V(C_5_H_7_O_2_)_3_ are usually used as source materials to synthesize VO_2_ by CVD method. Yakovkina et al. [75] prepared VO_2_ (M) thin film by metal–organic CVD (MOCVD). They used VO(acac)_2_-VO(C_5_H_8_O_2_)_2_ as the precursor and SiO_2_/Si(111) as the substrate. The flow rate ratio of argon/oxygen was controlled at 3–10 standard cubic centimeters per minute (sccm). The precursor source temperature was controlled in the range of 120–150 °C, and substrates were maintained at 390–490 °C, respectively. The whole growth process lasted for 30–120 min. The scheme of the CVD reactor for the growth of VO_2_ film is shown in Figure 8a. Compared with traditional CVD, MOCVD provides low temperature and low-pressure growth without post-growth annealing, and a shorter growth process. Sectional heating effectively avoided incomplete reactions of source materials. The morphologies of films with different grain sizes were obtained by adjusting the temperatures of the substrates, as shown in Figure 8b–e. The VO_2_ (B) phase mixed with the VO_13_ mesophase was obtained at low temperature (Figure 8b), and VO_2_ (M) monoclinic phase was obtained at a high temperature. CVD is a good way to synthesize not only pure phase VO_2_ (M) but also co-doped thin films. Guo et al. [76] reported Mo-Al-doped VO_2_ (B) thin films by CVD. V(C_5_H_7_O_2_)_3_ was employed as a V source mixed with Mo acetylacetonate ([CH_3_COCH=C(O)CH_3_]_2_MnO_2_) and Al acetylacetonate (Al(C_5_H_7_O_2_)_3_). The deposition processes were carried out in a three-zone furnace. The temperatures of precursor and substrate were 170 and 400 °C, respectively. The SEM image of Figure 8f shows large particles of Mo-Al-doped VO_2_ (B) thin films. The corresponding cross-sectional TEM images, SAED patterns and HRTEM images in Figure 8g–i indicate that the film is well crystallized along (001) direction.

#### 3.2.2. The Growth of VO_2_ Nanowires (NWs)

**Figure 9:**
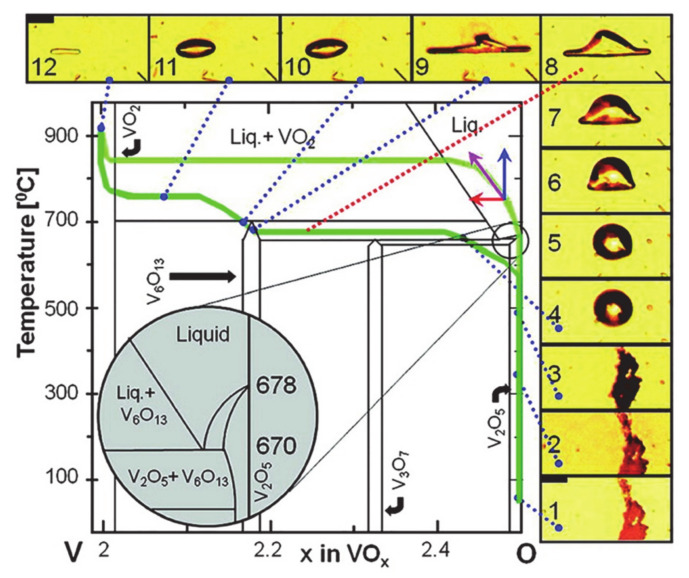
V–O temperature-composition phase diagram. Reproduced from [78], with permission from American Chemical Society, 2011.

In 2004, Guiton et al. firstly synthesized VO_2_ (M) NWs via CVD method by employing VO_2_ powders as the evaporation source [77]. The evaporation of VO_2_ usually requires a high temperature of more than 1000 °C. Therefore, the low melting point of V_2_O_5_ (690 °C) is commonly chosen as the reaction source to effectively decrease the deposition temperature. The phase diagram in Figure 9 summarizes the growth trajectories of VO_2_ (M) NWs prepared under different conditions [78]. During the CVD synthesis process, heating rate and oxygen loss rate are important factors that can determine the growth paths of VO_2_ NWs (green and light green paths in phase diagram). When the reaction source V_2_O_5_ powers are in a very low oxygen pressure environment and the heating rate reaches ~500 °C/min, VO_2_ NWs crystallize directly from the molten V_2_O_5_ precursor; see the light green path in Figure 9. On the contrary, under a lower heating rate, the precursor of V_2_O_5_ would first form V_6_O_13_ crystals at an insufficient temperature because of the lack of power to form VO_2_ nuclei and the slow loss of O. With the increase of temperature, V_6_O_13_ melts into droplets and becomes saturated. Finally, VO_2_ NWs are crystallized out.

**Figure 10:**
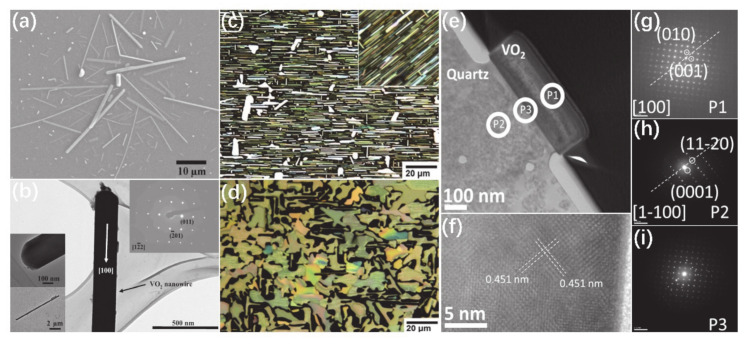
(**a**) SEM image of VO_2_ NWs. (**b**) TEM image of an individual VO_2_ NW. The insets are SEAD pattern and low-magnification TEM images of the growth front and the whole VO_2_ NW. Reproduced from [81], with permission from Elsevier, 2008. (**c**,**d**) The morphologies of the grown NWs and microplates. (**e**) TEM image of the cross-section of the interface between single NW and quartz substrate. (**f**) HRTEM image of VO_2_ NW. (**g**–**i**) The corresponding SAED patterns of the areas labelled with P1 (VO_2_), P2 (quartz), P3 (interface). Reproduced from [82], with permission from Springer Nature, 2014.

In recent years, the synthesis of VO_2_ NWs via CVD has been extensively studied [12,19,77,79,80]. Maeng et al. [81] reported a deposition process of VO_2_ NWs by using Si_3_N_4_/Si or molybdenum grid as the substrates. Figure 10a shows the SEM image of VO_2_ NWs. One can see that NWs are randomly distributed on the substrate and have different lengths. The SAED image in Figure 10b indicates that VO_2_ NWs were grown with different orientations. The random growth of NWs is directly related to the choice of substrates. Chen et al. [82] synthesized the horizontally aligned VO_2_ NWs on polished quartz with *x*-cut (1–100) direction, which can provide a dense growth mode of VO_2_ NWs. The deposition temperature has been controlled for growth of horizontally aligned NWs and microplates, as shown in Figure 10c,d. The HRTEM and SAED images indicated that the growth of NWs is along the (1–100)_quartz_ direction, as illustrated in Figure 10e–i. The temperature plays an important role in the control of the diameters and densities of VO_2_ NWs. VO_2_ NWs grown at low temperatures have small diameters and low density. On the other hand, high temperatures lead to the fusion of NWs and promote their lateral growth, resulting in the formation of a microplate nanostructure. The embedded VO_2_ microplates grown in the substrate have irregular shapes of sub-micron thickness and tens of a micron in size. Due to the elastic strain from the substrate, rich domain patterns are exhibited in the VO_2_ microplates, as shown in Figure 10d. One can see that substrate and temperature can directly affect the morphology of VO_2_.

**Figure 11:**
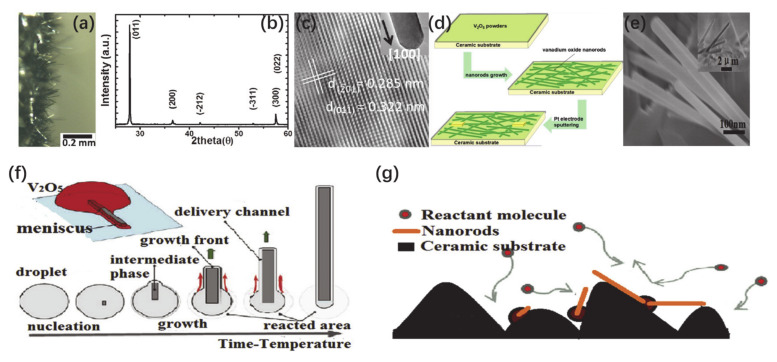
(**a**) Optical image of the side view of free standing VO_2_ NWs. (**b**) XRD pattern of the products. (**c**) HRTEM image of a VO_2_ NW; the inset shows the low-magnification TEM image of the growth front. Reproduced from [83], with permission from AIP Publishing, 2012. (**d**) Schematic diagram of the preparation of VO_2_ NRs. (**e**) SEM image of VO_2_ NRs, and the inset is the cross-sectional view. (**f**) The schematic diagram of the growth mechanism of free-standing VO_2_ NRs. (**g**) Nucleation and evolution of nanostructures in precursor droplets. Reproduced from [84], with permission from Elsevier, 2016.

In addition, Chen et al. [83] synthesized ultra-long, ultra-dense and free-standing VO_2_ micro/nanowires by using the unpolished rough surfaces of quartz substrates. Figure 11a shows the optical image taken from the side view, which indicates the VO_2_ NWs are free-standing and randomly dispersed on the substrate. The corresponding X-ray diffraction (XRD) pattern in Figure 11b shows that except the {0kl} peak of VO_2_ in-plane growth, there are abundant peaks of (200), (212), (311), (300), etc., which indicate the independent random orientation and non-epitaxial growth of VO_2_ NWs. The TEM image in Figure 11c demonstrates a smooth surface and a monoclinic structure of VO_2_ NWs. Similarly, Liang et al. [84] synthesized free-standing VO_2_ NWs by using rough surfaces of a ceramic substrate, as shown in Figure 11e. The preparation schematic diagram of VO_2_ NWs is shown in Figure 11d. V_2_O_5_ powder was employed as the evaporation source and was uniformly placed on a rough-surface substrate. The substrate for depositing products was placed in the range of 0.5 cm from the evaporation source. Besides, the growth mechanism of free-standing VO_2_ NWs was investigated, as described in Figure 11f,g. Under a high temperature, the V_2_O_5_ reaction source firstly melts into droplets. Such a liquid phase promotes the growth of nanostructure and then transports materials to the growth front. During the process of nanostructure growth from host droplets, a thin liquid film wets the nanostructure along the length direction to create a material transport channel for the growth of the NWs. Finally, the droplets are completely depleted and VO_2_ NWs are formed, as shown in Figure 11f. The rough surfaces of substrates (quartz, ceramic) provide favorable nucleation sites for NWs (as shown in Figure 11g). The dense nucleation sites lead to the formation of dense NWs. After the nucleation of NWs, the capillary force begins to appear at one end. The exposure of NWs to the reactive molecular atmosphere makes it easy to obtain the molecules needed for growth, so NWs can grow up to hundreds of microns in length.

**Figure 12:**
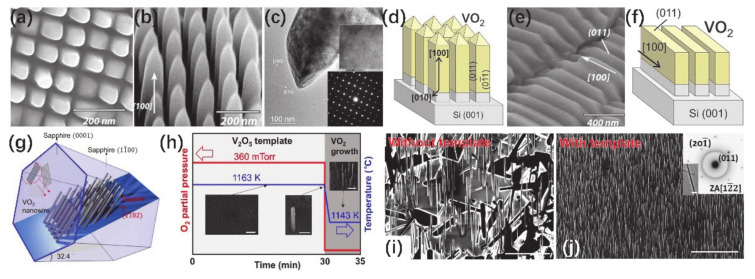
(**a**,**b**) SEM images of an ordered array of free-standing VO_2_ NWs from different perspectives. (**c**) TEM image of a VO_2_ NW and the corresponding SAED pattern. (**d**) Schematic diagram of the VO_2_ NWA grown on Si substrate. (**e**) SEM image of an ordered array of VO_2_ nanowalls and (**f**) a schematic diagram of an array of VO_2_ nanowalls. Reproduced from [85], with permission from AIP Publishing, 2018. (**g**) Diagram of the relative crystal orientation of the hexagonal prism of VO_2_ NW and sapphire crystal. (**h**) The design schematic diagram of the two-stage growth of VO_2_ nanoforest by controlling the oxygen pressure and substrate temperature with V_2_O_5_ droplets as template; the insets show SEM images of products at different growth stages. SEM of VO_2_ grown (**i**) without and (**j**) with V_2_O_5_ template. Reproduced from [86], with permission from American Association for the Advancement of Science, 2018.

Up to now, most VO_2_ NWs have been randomly distributed on the substrates and show random sizes and varied directions during the synthesis process. Mutilin et al. [85] reported the growth of single crystal VO_2_ NW arrays on Si (001) substrates with controllable size, spacing and orientation. A large-area double nanoimprint lithography (NIL) technique was employed to obtain periodic gratings printed on a Si surface. Figure 12a,b shows the top- and side-view morphologies of the synthesized VO_2_-on-Si nanostructures. The TEM image and SAED pattern from Figure 12c demonstrate the smooth surface and monoclinic structure of VO_2_ NWs. Moreover, horizontal VO_2_ NWs or nanowalls were also observed in some regions, as illustrated in Figure 12e. The insufficiency of the presswork at the second NIL stage resulted in the strips on Si, and then those VO_2_ NWs or nanowalls were grown on Si strips. Figure 12d,f shows the schematic diagrams of arrays of VO_2_ NWs and nanowalls, respectively. In addition, Wang et al. [86] proposed a novel approach for growing vertical-arranged VO_2_ NW forests via defect engineering. The design schematics of the VO_2_ NWs hybrid material system are depicted in Figure 12g. Sapphire (Al_2_O_3_) has been chosen as the appropriate substrate for the growth of aligned VO_2_ arrays. The freestanding strain-free VO*_x_* compound can be obtained by using the *r*-plane sapphire substrate. Choosing *r*-sapphire can also ensure a high nucleation rate and denser VO_2_ NW arrays. In this work, they designed a two-step, self-templated growth of VO_2_ NWs using V_2_O_5_ as the precursor, as shown in Figure 12h. The two steps of growth were as follows: (i) V_2_O_5_ droplets were grown as a template on *r*-plane sapphire; (ii) VO_2_ NWs were separated out from droplets to form neatly arranged NWAs. In the first stage, the carrier gas with a certain oxygen/argon volume ratio was used to create an oxygen-enriched environment to prevent the formation of vanadium with reducing (+3 and +4) valences. Regarding the other aspect, a much higher temperature (1163 K) than the melting point of V_2_O_5_ was employed to prevent its large-scale growth without leaving a molten droplet of V_2_O_5_ on the substrate. At the second stage of the growth, oxygen supply was suddenly interrupted and growth temperature was slightly reduced, which provided a greater supersaturation for VO_2_ growth. During this stage, the V_2_O_5_ droplet was a natural template for VO_2_ growth, so that more atoms could be adsorbed and vertically-arranged VO_2_ NW forests could be fabricated. As a contrast, the pure argon was used as the carrier gas, a continuous deoxidation process occurred at high temperatures and the growth was carried out without template. Top views of grown VO_2_ NW arrays without and with V_2_O_5_ template and two-stage process are compared in Figure 12i,j. The density of NWs varies greatly, and the reduction of redundant growth in the plane can be easily identified. Additionally, the inset of Figure 12j shows a TEM image and the SAED pattern of a single VO_2_ NW.

### 3.3. PLD

PLD can be dated back to the 1960s and was developed with the invention of a pulsed laser source and the discovery of high-temperature superconductivity. After decades of development, the range of materials that can be prepared by PLD is expanding greatly. PLD can be used to fabricate thin films ranging from monoatomic layers to quasi-bulk material. It occupies an irreplaceable position in fabrication of thin film [87]. In the PLD process, a high-power pulsed laser is focused on the surface of a target in a high vacuum chamber and the surface of the target is ablated to form plasma plume, which consists of target molecules and is deposited on the substrate to form thin film or other nanostructures. The rapid melting and evaporation of target under high-energy laser in a small area lead to all elements in the target evaporate at the same time. Therefore, it is easy to obtain multicomponent thin films with a stoichiometric ratio of target materials by the PLD method. In addition, PLD has the advantages of low temperature, high growth rate, in situ growth, etc. Moreover, the preparation parameters are easy to be adjusted, and there is no restriction on the types of target materials. Besides, it is convenient to prepare multilayers and superlattices by PLD.

#### 3.3.1. The Growth of VO_2_ Thin Films

**Figure 13:**
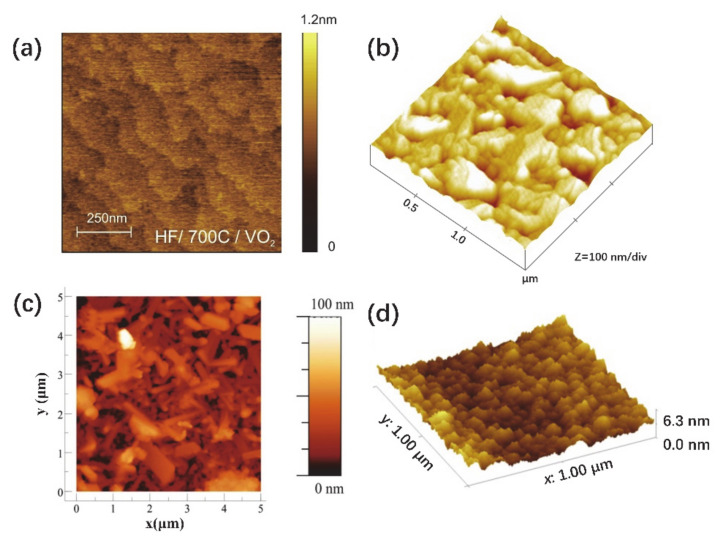
AFM images of VO_2_ thin film grown on different substrates. (**a**) Rutile TiO_2_ substrates. Reproduced from [88], with permission from AIP Publishing, 2014. (**b**) Al_2_O_3_ substrate. Reproduced from [90], with permission from AIP Publishing, 2011. (**c**) Glass substrate. Reproduced from [92], with permission from Elsevier, 2017. (**d**) Si/SiO_2_ substrate. Reproduced from [93], with permission from AIP Publishing, 2018.

Nowadays, a lot of studies have attempted to fabricate the VO_2_ thin film by PLD. TiO_2_ [88], Al_2_O_3_ [89,90], glass [91,92], Si/SiO_2_ [93], etc., are usually used as substrates in the PLD fabrication of a VO_2_ thin film. V_2_O_5_ [94], V_2_O_3_ [95] and VO_2_ [96] are usually used as the target materials. High-quality thin films can be obtained by adjusting the parameters of gas pressure, target-substrate distance, laser energy, deposition and annealing temperature. Figure 13 shows AFM images of VO_2_ thin films deposited on TiO_2_, Al_2_O_3_, Si/SiO_2_ and glass substrates. As is well known, the high-temperature metal phase of VO_2_ (R) has the same crystal structure and space group as rutile TiO_2_; thus, TiO_2_ is usually used as a substrate in the deposition of VO_2_. Figure 13a shows the AFM image of a VO_2_ thin film deposited on TiO_2_ (001) [88]. Here the TiO_2_ substrate was etched by HF and annealed at 750 °C in an oxygen environment to form terraces. Terraced VO_2_ thin film with atomic-level smoothness was epitaxially grown on the TiO_2_ substrate. On the other hand, VO_2_ is monoclinic and pseudo-hexagonal at room temperature. Therefore, hexagonal Al_2_O_3_ is also an ideal substrate for the growth of an epitaxial VO_2_ thin film, and the lattice mismatch is within a few percentage points [97]. As a contrast to traditional deposition techniques with high-temperature deposition, a VO_2_ thin film can be fabricated on *c*-cut (0001) Al_2_O_3_ substrate at room temperature by using PLD [90]. The surface of a VO_2_ thin film is shown in Figure 13b, and it indicates that the RMS roughness of VO_2_ thin films is about 4.5 nm [90]. Glass substrates have advantages in cost and industrial applications, especially in smart windows. Previous work also has attempted to fabricate the VO_2_ thin film on a glass substrate [92], as shown in Figure 13c. For the deposition of VO_2_ on a glass substrate, different VO_2_ phases (e.g., M1 phase and B phase) can be obtained by adjusting the deposition temperature [92]. From the AFM image of VO_2_ thin film in Figure 13c, the roughness is about 10 nm. As the most commonly used substrate, Si/SiO_2_ substrate is usually used for depositing the VO_2_ thin film [93]. The VO_2_ thin film deposited on Si/SiO_2_ substrate exhibits dense and continuous crystalline nanostructures, as shown in Figure 13d. The roughness of as-grown VO_2_ thin films is about 0.671 nm. This demonstrated that the VO_2_ thin film deposited on Si/SiO_2_ substrate has a good surface morphology. Importantly, in this work, the results have demonstrated that the oxygen partial pressure has significant effects on the microstructures of the films [93].

#### 3.3.2. The Growth of VO_2_ Low-Dimensional Structures (LDSs)

**Figure 14:**
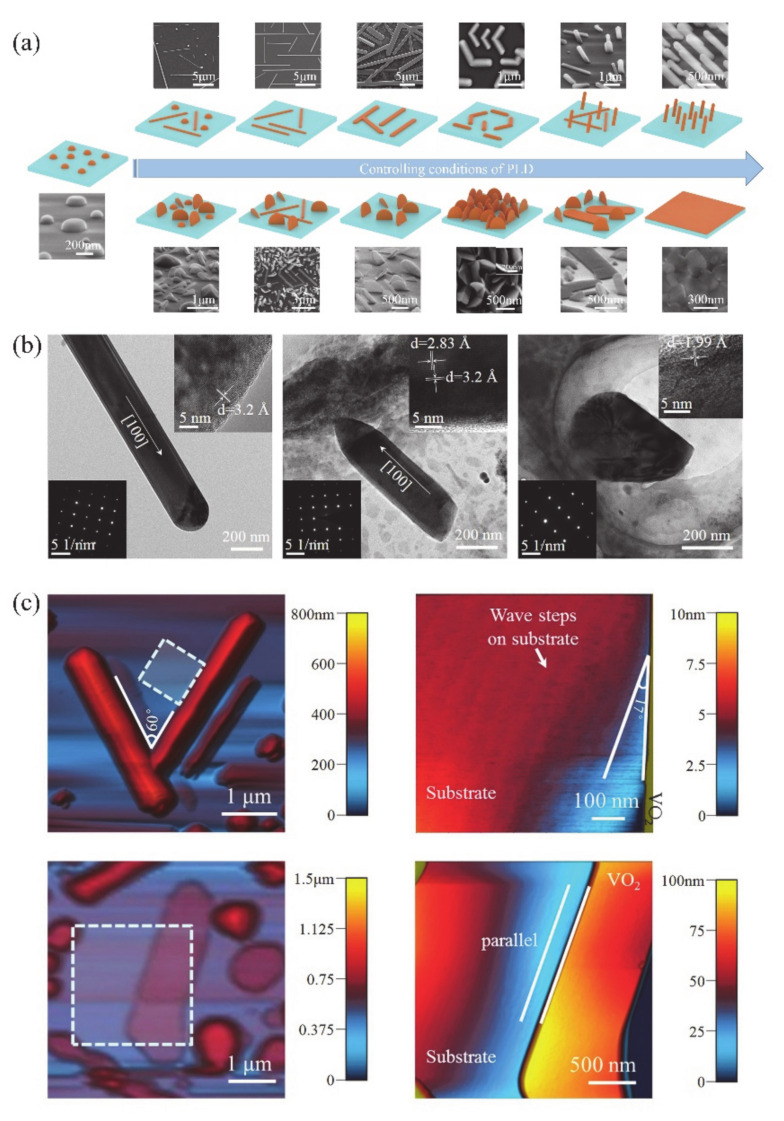
(**a**) Schematic diagrams and corresponding SEM images of as-deposited low-dimensional structures (LDSs). (**b**) TEM images of representative NW, vertically-grown NR and nanoplatelets, and the insets show the corresponding HRTEM images and SAED patterns. (**c**) AFM and high-resolution AFM images of NWs and laterally grown NPs. Reproduced from [16], with permission from Royal Society of Chemistry, 2018.

As is known to all, PLD is an excellent method for depositing thin film structures. Interestingly, under certain deposition conditions, it is also an effective approach to fabricate LDSs. In our previous work, by controlling the deposition conditions (e.g., substrate, oxygen pressure, deposition and annealing temperatures, pulse count and energy of laser, the distance between the target and substrate, etc.), numerous LDSs of 0D NDs, 1D NWs, NBs and NRs; two-dimensional (2D) nanoplatelets; ultra-thin films; and also various mixed structures were fabricated [16]. Figure 14a shows the schematic diagrams and corresponding SEM images of as-deposited LDSs. In this work, the substrates of (0001) Al_2_O_3_, (10–10) Al_2_O_3_, (0001) SiO_2_ and Si/SiO_2_/Pt were employed to deposit the VO_2_ structures. The results demonstrate that the NWs and NBs are favored to grow on (0001) Al_2_O_3_ and (001) SiO_2_ substrates, respectively. The angle between the two NWs grown on (0001) Al_2_O_3_ substrate is 60° (or 120°). On the other hand, the nanoplatelets tended to grow along the step-terraces of the substrate (Figure 14c). Besides, the length/diameter ratio of NW can be conveniently controlled by adjusting the oxygen pressure. Interestingly, vertically-grown NWs and nanoplatelets were also obtained by controlling the pulse number of the laser and annealing treatment process. HRTEM images and SAED patterns indicate that the NW, vertically-grown NW and nanoplatelets have high crystallinity, as shown in Figure 14b. Moreover, the temperature-dependent Raman spectra and XRD patterns reveal that the MIT properties of VO_2_ LDSs and the mismatch strain between LDSs and substrate lattice are closely related. Therefore, PLD can be employed as a simple and controllable technique to fabricate VO_2_ LDSs.

### 3.4. Sol–Gel Method

The sol–gel method can be traced back to the middle of the nineteenth century [98], also known as the chemical solution deposition (CSD) or liquid-phase sol–gel (solution-sol–gel, SSG) method. The inorganic polymer sol–gel method is one of the most commonly used material synthesis technologies. In general, metal alkoxides are dissolved in appropriate organic solvents, and then undergo hydrolysis and polymerization to form sol. Sol further loses most of the solvent to form a gel with a continuous three-dimensional network structure. Finally, ultrafine powders, fibers and films can be prepared after heat treatment. Since the invention of sol–gel method, it has developed rapidly and has been widely employed for preparing various structures of material, e.g., bulk, powder, porous material, fibers and films. The sol–gel method has the merits of low cost and large area deposition. Most importantly, the sol–gel method is a chemical synthesis approach, so it can easily control the stoichiometry and the element doping.

**Figure 15:**
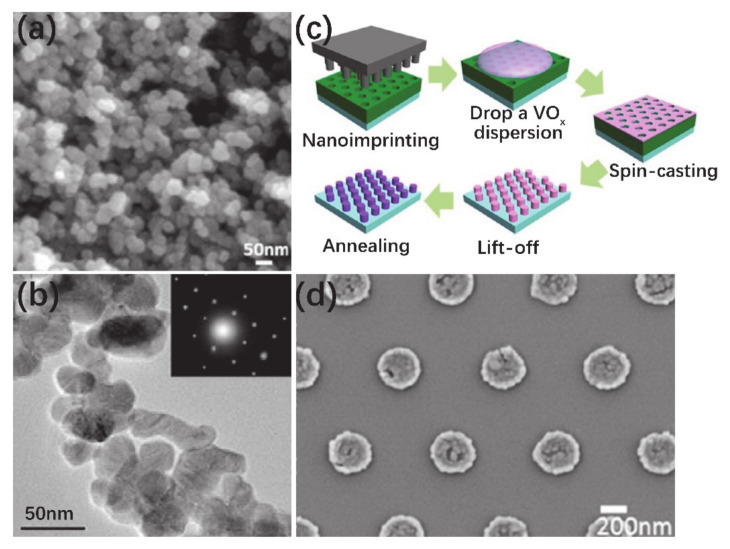
(**a**) SEM image of VO_2_ nanopowders and (**b**) TEM image of VO_2_ nanopowders; the inset is the SAED pattern of an individual particle. Reproduced from [106], with permission from Elsevier, 2011. (**c**) Schematic diagram of VO_2_ nanostructure fabricated by nanoimprint lithography. (**d**) SEM image of VO_2_ nanostructure arrays. Reproduced from [107], with permission from American Chemical Society, 2014.

It was reported decades ago that VO_2_ thin films can be synthesized via sol–gel method [99,100,101,102]. The synthesis of VO_2_ by the sol–gel method usually consists of spin-coating or dip-coating and an appropriate annealing process [102,103]. In recent years, VO_2_ thin films [99,104,105], nanopowders [106] and nanopillars [107] have been successfully synthesized. Importantly, sol–gel can be conveniently used for synthesizing doping VO_2_ structures, e.g., W-doped [103,108], Au-doped [109] and Mo-doped [110] thin films. Wu et al. [104] coated the sol solution on an Al_2_O_3_ (0001) substrate and obtained high-quality VO_2_ thin film after an appropriate annealing process with a uniform grains size about 100 nm. As mentioned above, VO_2_ NPs of small size have great advantages in the application of smart windows, and VO_2_ NPs with an average size of 26 nm have been prepared by hydrothermal method (Figure 3b) [50]. By combining the sol–gel and hydrothermal methods, the size of VO_2_ NPs is expected to be further reduced. Ji et al. [106] synthesized high-quality single crystal VO_2_ nanopowders by using V_2_O_5_·nH_2_O sol as a vanadium precursor, which was prepared by the dissolution reaction of V_2_O_5_ in H_2_O_2_. By pre-reduction and hydrothermal treatment, the VO_2_ nanopowders with an average size of less than 20 nm were obtained. Figure 15a,b shows the SEM and TEM images of VO_2_ nanopowders, respectively. The inset of Figure 15d illustrates the SAED pattern for one nanoparticle, which indicates that the monocrystalline VO_2_ nanopowders were obtained. Besides, VO_2_ nanostructure arrays also can be fabricated by using the sol–gel method assisted by templates (polystyrene spheres or nanoimprint lithography) [107,111]. VO_2_ nanoparticle, nanodot and nanonet arrays have been fabricated by the nanosphere lithography technique in different growth conditions [111]. Similarly, Paik et al. [107] used nanoimprint lithography to fabricate a VO_2_ nanostructure; the schematic diagram is illustrated in Figure 15c. Firstly, the size and the shape of nanoimprint patter were designed, and the nanoimprint lithography was employed to realize the corresponding polymer resist template. Then, the colloidal nanocrystals were spin-coated on top of the patterned template. After that, the polymer resist was lifted off and the nanopillars were successfully prepared after annealing process, as shown in Figure 15d.

On the other hand, as we mentioned before, it is a challenge to synthesize high quality epitaxial VO_2_ films due to the multivalence states of V element (V^2+^, V^3+^, V^4^^+^, or V^5+^). However, the sol–gel method (or chemical solution) can conveniently modulate the oxygen stoichiometry in vanadium oxide. In previous work, a VO_2_ thin film with enhanced MIT properties was synthesized by using a moisture-assisted chemical solution method [112]. In order to obtain high performance VO_2_ films, it is very important to prevent the formation of V^5+^ or V^3+^ during the growth process. Liang et al. [112] introduced the moisture in synthesis process and found that the moisture can significantly inhibit the formation of V^5+^ or V^3+^. Intrinsically, moisture is employed to provide oxygen vacancy in the preparation of oxides [113], which is expected to inhibit the formation of V^3+^ ions. Meanwhile, moisture is not active enough to oxidize V^4+^ ions to V^5+^ ions. Therefore, the oxygen stoichiometry of VO_2_ can be precisely control and then the enhanced MIT performance of a VO_2_ thin film can be obtained.

### 3.5. Other Methods

In addition to the methods described above, there are several other methods also can be used for fabricating VO_2_ nanostructures, such as magnetron sputtering [8,114,115], electrospinning [42] and MBE [116,117,118].

#### 3.5.1. Magnetron Sputtering

**Figure 16:**
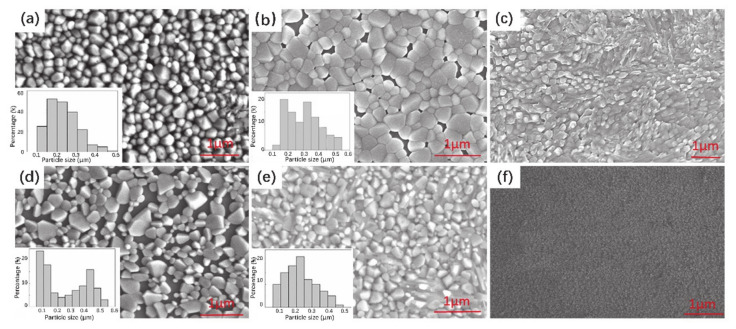
SEM images of VO_2_ thin films with different oxidation conditions: (**a**) 400 °C, 2 sccm, (**b**) 425 °C, 2 sccm, (**c**) 410 °C, 2 sccm, (**d**) 425 °C, 1 sccm, (**e**) 425 °C, 5 sccm and (**f**) 390 °C, 2 sccm. Reproduced from [121], with permission from AIP Publishing, 2017.

Magnetron sputtering began in the 1970s and developed rapidly because of its compatibility with microelectronic manufacturing technology [119,120]. In magnetron sputtering technology, incident ions (charged particles) obtain high energy under high-voltage electric fields in a vacuum and bombard the target surface. Target atoms get enough energy from the high-energy incident ions and escape from the target surface, and then deposit on the substrate to form a thin film. Differently from ordinary ion sputtering, the movement tracks of high-energy ions are controlled by a magnetic field, which can significantly promote the lifetime of incident ions and sputtering rate. Magnetron sputtering is a common technology for the preparation of thin films. Yu et al. [121] reported the fabrication of VO_2_ thin films with two steps. In the first step, pure vanadium thin films were deposited on a sapphire substrate by DC magnetron sputtering and then oxidized in a pure oxygen environment. Figure 16a–f displays the SEM images of the VO_2_ thin films with several oxidation conditions; the insets show the grain size distribution. Obviously, the oxidation conditions, e.g., temperature and oxygen flow rate, play important roles in the grain size, shape and arrangement [122].

#### 3.5.2. Electrospinning

**Figure 17:**
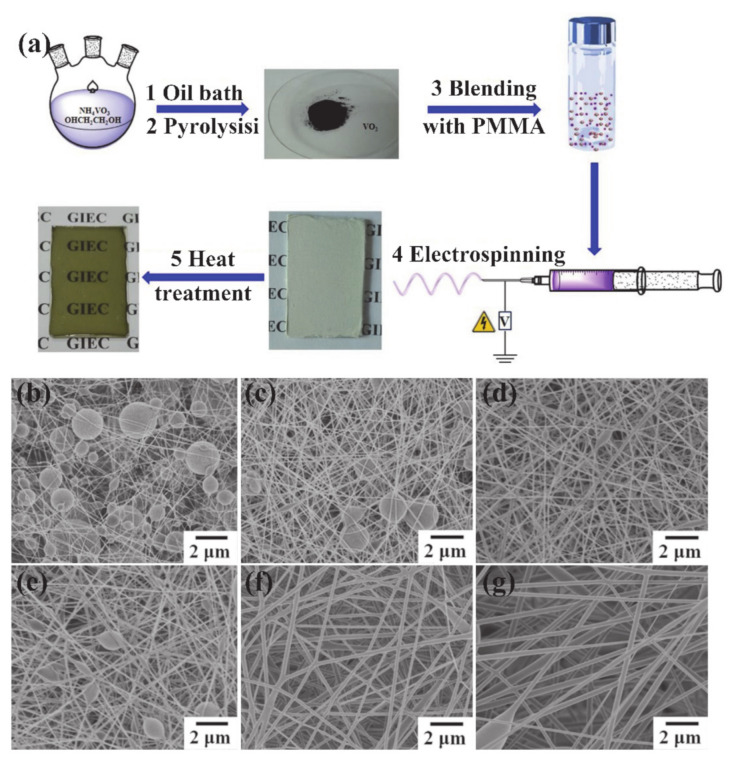
(**a**) Schematic diagram of the fabrication process of the VO_2_ film via electrospinning technique. (**b**–**g**) SEM images of the PMMA nanofibrous mats with different concentration (*w*/*w*): (**b**) 10%, (**c**) 12%, (**d**) 14%, (**e**) 16, (**f**) 18% and (**g**) 20%. Reproduced from [42], with permission from Elsevier, 2017.

Electrospinning is a method to form fibers from a molten polymer or polymer solution under the application of a high voltage electrostatic field. In a high voltage electrostatic field, the charged molten polymer or polymer solution undergoes a series of static spinning processes, such as spraying, stretching, splitting, solvent evaporation or solidification, and finally forms precursor nanofibers. Electrospinning is one of the most important methods for preparing nanofibers. Synthesis of V_2_O_5_/poly (vinyl acetate) by electrospinning followed by thermal treatment was reported dozen years ago [123]. Recently, Lu et al. [42] successfully prepared opaque VO_2_-based composite nanofibers on glass substrates by electrospinning technique. Then, a VO_2_ (M) thin film was obtained after heat treatment. Such a thin film of woven fibers could be used in the application of transparent thermochromic smart windows. Figure 17a describes the detailed procedures of transparent VO_2_ thin films via the electrospinning technique. The procedures include synthesis of VO_2_ NPs (stages 1 and 2), preparation of PMMA-VO_2_ electrospun composite nanofiber (stages 3 and 4) and heat treatment (stage 5). From Figure 17b–g, the morphologies of PMMA nanofibers vary with the concentration of polymer, which is due to the different viscosities and conductivities of the solutions [124].

#### 3.5.3. Molecular Beam Epitaxy (MBE)

MBE is a technique for epitaxial growth of single crystal thin films with several atomic layers. The emergence of the MBE technique has had a tremendous impact on materials science. MBE equipment works in an ultra-high vacuum environment to prevent the effects of impurities. There are various elemental source furnaces and substrate heaters in the chamber with an ultra-high vacuum. Atoms or molecules in the heated source furnace will be ejected from the source furnace when opening the source furnace switch valve. Due to the van der Waals forces, the ejected atoms or molecules will combine with each other on the surface of the substrate to form a stable ultrathin film. For the fabrication of the VO_2_ films by using MBE, the challenge comes from the control of V and O beam flux in stoichiometric. It is difficult to control the evaporation of V source due to its high melting point and low saturated vapor pressure. In addition, V has numerous chemical valence states and its chemical valence is sensitive to the oxygen pressure. Fu et al. [116] fabricated a high-quality, pure M phase VO_2_ thin film by precisely controlling the V and O stoichiometric ratio in MBE deposition. The obtained VO_2_ (M) epitaxial film on Al_2_O_3_ (0001) substrate had a smooth surface, uniform thickness and uniform phase transformation performance [116]. Besides, Paik et al. [117] prepared ultrathin VO_2_ thin films by the MBE method on TiO_2_ (001) substrate. The thickness of VO_2_ thin film can be decreased to 1.5 nm. MBE is an excellent way to grow an ultra-thin VO_2_ thin film compared with other techniques.

### 3.6. Summary

The strategies for the growth of VO_2_, containing the hydrothermal method, CVD, PLD, sol–gel, magnetron sputtering, electrospinning, MBE, etc., are introduced in this section. The hydrothermal method is a useful method for fabricating the polymorphs of VO_2_ (e.g., VO_2_ (A), VO_2_ (B), VO_2_ (C), VO_2_ (D), VO_2_ (P) and VO_2_ (M)) and various LDSs of VO_2_ (e.g., NPs, NWs, NRs, nanorings and microsheres). The advantage of a simple process and high yield made hydrothermal method become a popular approach for the growth of VO_2_. CVD is mainly used for the growth of VO_2_ NWs. Compared with the hydrothermal method, the VO_2_ NWs synthesized by CVD usually have a larger length-to-diameter ratio. Besides, CVD can be also used for depositing VO_2_ thin films by controlling the deposition conditions. For the growth of VO_2_ thin films, PLD, magnetron sputtering and MBE are popular methods to realize the high-quality film. MBE is a technique for the epitaxial growth of ultrathin VO_2_ films. PLD is not only a simple way to deposit a VO_2_ thin film, but also an effective method to fabricate various LDSs, e.g., NDs, NWs, NBs, NRs and nanoplatelets. Sol–gel and electrospinning technologies can be used to synthesize VO_2_ thin films and nanofibers, respectively. To be clearer, the advantages and weaknesses of the mentioned preparation methods of VO_2_ have been listed and compared in Table 2.

## 4. Properties and Related Applications of VO_2_

Due to the excellent MIT behaviors and near-room-temperature *T_c_*, VO_2_ has great potential applications in electronics (e.g., electronic switches, field-effect transistors and memories), optical devices and various sensors. In recent years, researchers have explored the applications of VO_2_ by utilizing the unique response characteristics of VO_2_ to external stimuli. Here, we briefly summarize the latest application progress of VO_2_.

### 4.1. Electrical Devices

#### 4.1.1. Electronic Switch

**Figure 18:**
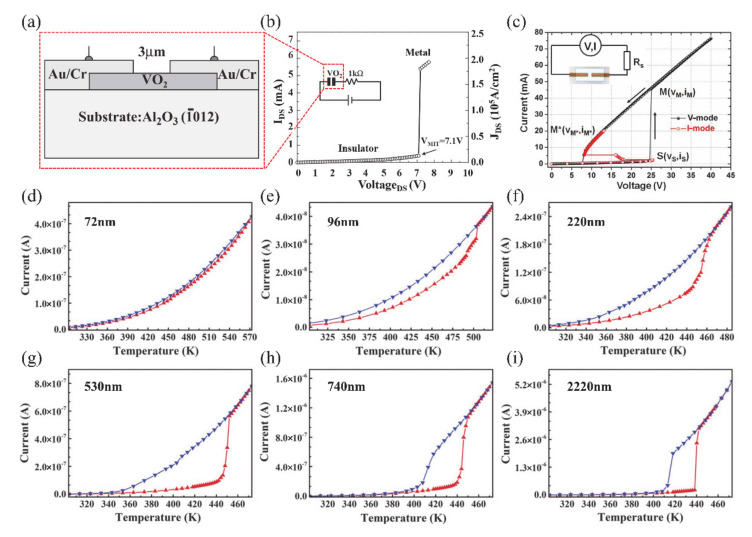
(**a**) Test circuit diagram of VO_2_ thin film MIT monitored by switching voltage pulse. (**b**) MIT driven by a DC voltage in VO_2_ thin film. Reproduced from [7], with permission from Elsevier, 2005. (**c**) I–V curve of a two-terminal VO_2_ switch, M* means that when the voltage is reduced, VO_2_ reaches point M* where VO_2_ transforms back to an insulator. Reproduced from [125], with permission from IOP Publishing, 2010. (**d**–**i**) Temperature-current hysteresis loops with different widths of VO_2_ (A) NW, (**d**) 72 nm, (**e**) 96 nm, (**f**) 220 nm, (**g**) 530 nm, (**h**) 740 nm and (**i**) 2220 nm. Reproduced from [20], with permission from Royal Society of Chemistry, 2016.

The huge change of conductance during MIT is an important property of VO_2_, which makes it a great potential material for electronic switches [7,125,126,127,128]. Generally, the electronics switches based on VO_2_ can be driven by temperature or by applying a static current [125] and a static voltage [7,127]. The electronic switch based on VO_2_ has good performance in speed and broadband operation [125]. The connection and disconnection of the circuit can be controlled by adjusting the metal or insulating phase of VO_2_ when it is connected in series to the circuit. Most electronic switches are operated based on voltage-driven MIT of VO_2_. Stefanovich et al. [126] designed a VO_2_-based electronic switch device and reported that the MIT occurred at a threshold voltage of 2 V. The experimental results demonstrated that the phenomenon of switching no longer occurred when the temperature was higher than *T_c_*. Therefore, temperature plays an important role in VO_2_ electronic switching. Boriskov et al. [127] studied the I–V characteristics of VO_2_ switching devices at different temperatures. Different voltages are required to trigger MIT when the ambient temperature is changed. Figure 18a illustrates the cross-sectional diagram of a VO_2_ two-terminal switching device, and the corresponding electronic switching characteristics of the device are shown in Figure 18b [7]. In addition, Crunteanu et al. [125] studied voltage-mode and current-mode VO_2_-based switches, as shown in Figure 18c. They demonstrated that current-driven VO_2_-based switches operated more than 260 million cycles without failure; that is a longer lifetime than in the voltage-driven mode (breakdown at about 16 million cycles). Thus, the integration of VO_2_ has great potential in device applications with large numbers of stable and repeatable switching cycles. On the other hand, as is known to all, temperature is a facile way to achieve the resistance switching of VO_2_. Therefore, the corresponding resistance switching is consistent with MIT behavior. As a strongly correlated electron material, the MIT behaviors of VO_2_ are sensitive to the size and surface effects. In our previous work, the two-terminal switching devices based on single crystalline VO_2_ (A) NWs with different sizes were reported [20]. Due to the size and surface effects, the hysteresis loops, MIT temperatures and resistance switching of VO_2_ (A) with different widths showed great differences, as shown in Figure 18d–i. When the width of NWs is less than the critical size, the first-order phase transition even transforms via high-order, continuous phase transition. Therefore, it presents abundant resistance switching states to meet various application requirements.

#### 4.1.2. Field-Effect Transistor (FET)

Field-effect transistors (FETs) are conductive devices based on the early triodes and are controlled by the gate voltage. Compared with triodes, FETs have the advantages of high input impedance, low temperature effect, low noise, low power consumption, high switching speed and low production process difficulty. Among all the FETs, the metal–oxide-semiconductor FET (MOSFET) is one of the most widely used semiconductor devices [129]. MOSFET generates current by carrier diffusion and drift. In recent years, silicon-based FETs have become an essential electronic unit in modern semiconductor technology. However, the inherent size limitation of silicon makes it unsuitable for the manufacture of smaller FET devices. The sub-threshold swing should not be lower than the limit of 60 mV/dec. Otherwise, the power consumption, switching rate and integration of FET devices will be seriously influenced. Therefore, it is an urgent need to find alternatives for silicon materials. MIT materials are considered as promising channel materials for nanoscale FETs. Compared with traditional silicon-based materials, MIT materials have larger changes of carrier concentration during MIT [130,131]. Among the FETs based on MIT materials, VO_2_-based FETs have been widely studied due to their ultrafast MIT speeds and large changes of carrier concentration. Hormoz et al. [132] demonstrated that the concentrations of free carriers induced by MIT in VO_2_ are several orders of magnitude larger than those in silicon. Therefore, the VO_2_-based FET can easily overcome the carrier-transit-time limitation of traditional MOSFET.

**Figure 19:**
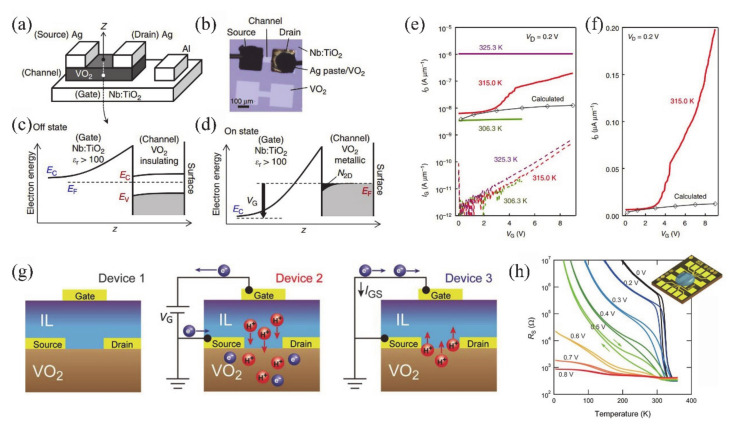
(**a**–**d**) Device structures and (**a**) a schematic illustration, (**b**) an optical micrograph and (**c**,**d**) the band diagrams of the fabricated transistor. (**e**) Transfer characteristics of the VO_2/_Nb:TiO_2_ transistor with the drain voltage of 0.2 V. (**f**) Transfer characteristics at 315.0 K in the linear scale. Reproduced from [135], with permission from Springer Nature, 2015. (**g**) Schematics of VO_2_ FET based on IL gate in the pristine state (Device 1), with gate-induced protonation (Device 2) and after returning to the pristine state (Device 3). (**h**) Temperature dependence of the channel resistance about the VO_2_ FET based on ILs gate at various gate voltages. Reproduced from [138], with permission from John Wiley and Sons, 2016.

Recently, some attempts have been carried out to combine VO_2_ thin films/NWs with solid-state or ionic liquid (IL) electrolyte gate dielectrics [4,5,6,40,133,134,135,136,137,138]. The SiO_2_, TiO_2_, Al_2_O_3_/SiO_2_ and oxide/organic hybrids were usually used as solid-state gate dielectrics [5,133,134,135,136]. For the VO_2_-based FETs, the controllable MIT behavior plays an important role on the design of electronic devices. Yajima et al. [138] observed a positive-gate-bias-controlled MIT behavior of VO_2_ near the transition temperature with the high-permittivity TiO_2_ as gate dielectric. The schematic diagram and corresponding optical images of the device are shown in Figure 19a,b. The *n*-type Nb-doped titanium dioxide (Nb:TiO_2_) single crystal was used as the back gate electrode to realize the reverse Schottky gate geometry, as displayed in Figure 19c,d. In this inverse Schottky grid geometry, high permittivity and high breakdown voltage can be achieved in the depleted single-crystal Nb:TiO_2_ dielectric at the interface. They observed that a large number of electrons were accumulated through the modulation of high-permittivity TiO_2_ gate (see Figure 19e,f). Besides, Wei et al. [136] reported a VO_2_-based FET by using high-*k* oxide Ta_2_O_5_/organic parylene-C hybrid as the gate dielectric layer. They found a significant hole–electron asymmetry that is related to the suppression of *T_c_* of VO_2_. It was proposed that the MIT of VO_2_ may be induced by the electrostatic field. Based on this, Abbas et al. [5] used Al_2_O_3_/SiO_2_ hybrid gate dielectrics to control the MIT behavior of VO_2_ at room temperature. The carrier concentration in VO_2_ devices raises with the increase of gate electrostatic effect. This was attributed to the electronic transition under the electrostatic effect [5]. However, in general, the regulation of solid-state gate cannot achieve the desired results [136,139,140]. It can be attributed to the insufficient quality of gate dielectrics layer and interface between gate and channel, which leads to the poor electric field effect and cannot provide enough electron density accumulation for MIT in VO_2_ [136].

On the other hand, the IL dielectric gate, which can produce a stronger electric field than the solid-state dielectric gate, has become a popular gate of VO_2_-based FETs [6,40,137,138]. In electrolyte gating, a high electric field in an electric double layer (EDL) at the interface between electrolyte and channel material can lead to the accumulation of carrier density exceeding 10^14^ cm^−2^, which is more than 10 times larger than that of traditional gate modulation [138]. This accumulation of high carrier density allows the induction of electron phase transition in the relevant electronic oxides. Previous works have demonstrated the ILs-gate-induced reversible MIT in VO_2_ [141], and sometimes there is no electrostatic effect in VO_2_ except doping [142]. Moreover, once the defects are formed, the MIT should become irreversible [6]. Shibuya and Sawa [138] reported a reversible MIT of VO_2_, which was achieved by protonation reaction under gate modulation. Figure 19g shows the working process of the VO_2_ FET based on ILs gate, which is mainly the migration of H^+^ from ILs between ILs and VO_2_ channel under ILs gate electric field [138]. With gate-induced protonation, the H^+^ from ILs migrates into VO_2_, and the chemical reaction is VO_2_ + γH^+^ + γe^−^ → H_γ_VO_2_. After restoration to the pristine state, the chemical reaction is H_γ_VO_2_ → γH^+^ + VO_2_ + γe^−^, and H^+^ from the VO_2_ channel migrates into the ILs. As shown in Figure 19h, a reversible MIT of VO_2_ is demonstrated from the temperature-dependent plate resistance (*R*_s_) in VO_2_-based ILs FET at different gate voltages. What is more, Jo et al. [143] used solid-state proton electrolyte to achieve a reversible MIT of VO_2_ at room temperature. A large number of H^+^ ions were effectively injected into VO_2_ channel under gate bias voltage without oxygen defects. The H^+^-induced MIT of VO_2_ is due to the large modulation of the out-of-plane lattice parameters by the H^+^-induced chemical expansion.

**Figure 20:**
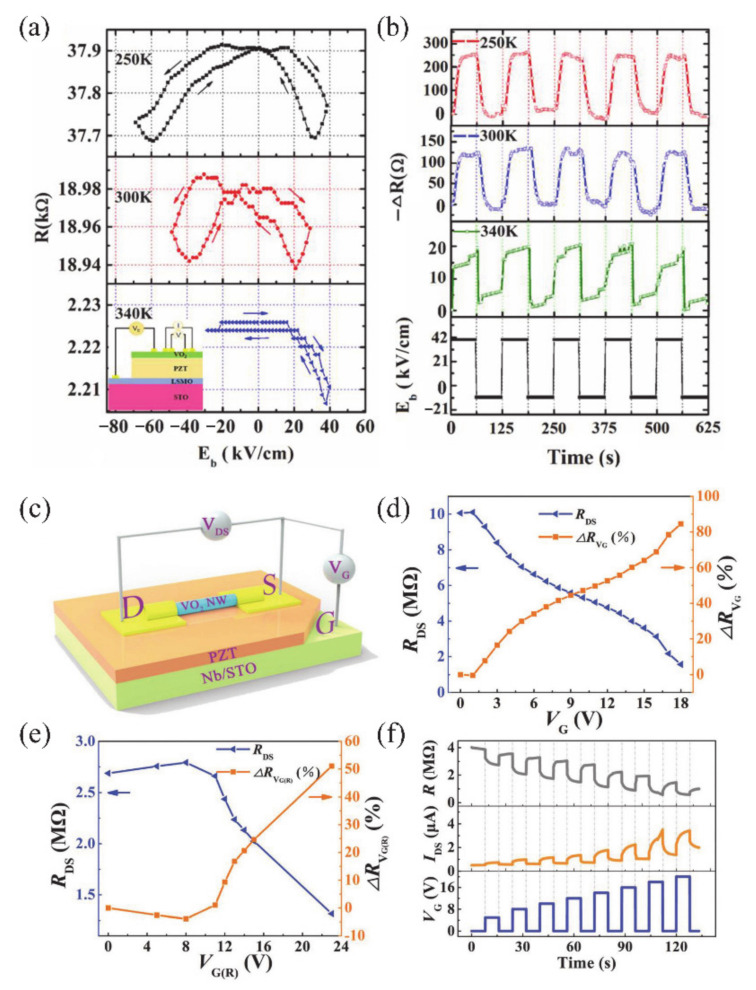
(**a**) Continuous and (**b**) pulsed gate electric field dependences of the resistance in a VO_2_ thin film under 250, 300 and 340 K; the inset of (**a**) shows the VO_2_/ferroelectric heterostructure device. Reproduced from [145], with permission from AIP Publishing, 2019. (**c**) Schematic diagram of the fabricated VO_2_ NW-FeFET. (**d**) The resistance change of the VO_2_ NW-FeFET ranging from 0 to 18 V. (**e**) Resistance change of the VO_2_ NW-FeFET ranging from V_G(R)_ = 0 to V_G(R)_ = 23 V; here the V_G(R)_ denotes that the gate voltage is applied and then removed. (**f**) Time-dependent current and resistance evolutions of the VO_2_ NW channel under a series of gate pulses from 5 to 20 V. Reproduced from [147], with permission from Royal Society of Chemistry, 2020.

Moreover, ferroelectrics also are popular dielectric materials in recent years. The ferroelectric materials have excellent properties—a strong polarization response, high-speed switching and a nonvolatile nature. In the ferroelectric gate FET (FeFET), the polarization orientations can be easily tuned by an applied electric filed. Moreover, the remnant polarization in the ferroelectric means the polarization state can be maintained even if the electric field is removed. In recent years, studies on VO_2_-based FeFET have been reported. The VO_2_/ferroelectric heterojunctions have been fabricated and different polarization states in ferroelectrics can be tuned through the electric field. Therefore, different strain states are induced at the interface of VO_2_/ferroelectric and the multi-resistance state of VO_2_ is realized [144,145,146]. The MIT behavior of VO_2_ is sensitive to the strain effect so that multi-resistance states are obtained. These properties of VO_2_ make it have a potential application in strain sensors (detailed in the next section). Zhang et al. [145] reported a VO_2_/ferroelectric thin film heterostructure device and investigated the piezoelectric control of resistance switching in a VO_2_ thin film, as the device shown in the inset of Figure 20a. Under the electric field, the piezoelectric effect of ferroelectric thin film introduces the lattice strain at the interface of a VO_2_/ferroelectric thin film, and leads to the reversible resistance change of VO_2_. The modulation of electric field at different temperatures shows that the lattice strain at the interface of VO_2_/ferroelectric will degenerate at a high temperature (close to *T_c_*) (Figure 20a,b). Therefore, a lower temperature is more conducive to the piezoelectric control of resistance switching in VO_2_. In previous work, the VO_2_ NW-based FeFET was fabricated based on VO_2_ NW and a ferroelectric thin film of Pb(Zr_0.52_Ti_0.48_)O_3_ (PZT), with the VO_2_ NW as a conducting channel and PZT as a gate dielectric layer (Figure 20c) [147]. In this work, the conductance of the VO_2_ NW channel could be modulated by the ferroelectric gate. The resistance change was up to 85% under the gate voltage of 18 V (electric field of ~0.75 MV cm^−1^). Moreover, a large resistance change of 50% was still observed even though the system under the zero gate voltage. It was attributed to the remnant polarization in the ferroelectric thin film being also able provide the similar equivalent electric field for the channel of VO_2_ NW. Additionally, multiple resistive states could be realized when the pulsed gate voltage was applied for the device (Figure 20d–f). This work offers a potential strategy for developing the nonvolatile device based on VO_2_ FETs.

#### 4.1.3. Memory Device

**Figure 21:**
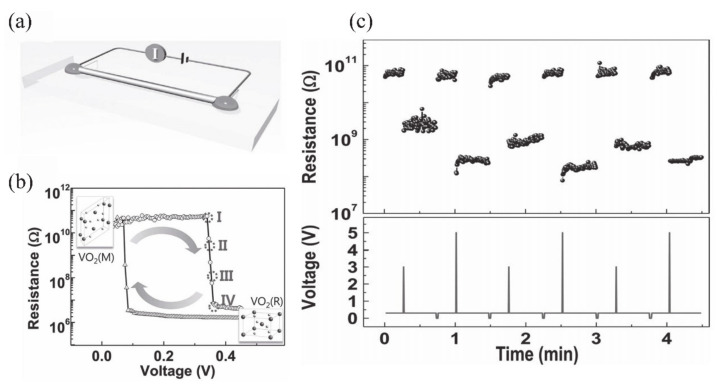
(**a**) A schematic of the memristor device. (**b**) The resistance–voltage (R–V) hysteresis curve. (**c**) Demonstration of the information storage. Reproduced from [10], with permission from John Wiley and Sons, 2013.

Generally, an electric field is considered as another effective way to drive MIT behavior, other than temperature. Hysteresis usually exists during the MIT and is usually attributed to the different phase transition paths during the cooling and heating processes. Such a hysteresis is affected by strain, doping and lattice defects during MIT process. VO_2_ can receive the mismatch strain from the substrate when it is grown on a substrate. Besides, VO_2_ is often deposited on the substrate in most electronic devices, resulting in a large external strain during MIT. As a result, the hysteresis leads to the metallic phase of VO_2_ being retained at a relatively low temperature (or applied voltage). Therefore, VO_2_ can be developed into another potential application in memory devices [12,148,149,150,151]. Combining the advantages of an independent all-oxide cantilever beam and the hysteresis characteristics of VO_2_, Pellegrino et al. [151] realized a multi-state memory with two-terminal current control. They used VO_2_/TiO_2_ free-standing thin film heterostructures via a Joule self-heating process and achieved multistate memory devices nearby the *T_c_* of 70 °C. The memory effect is caused by controlling the formation and growth of metal clusters in the micrometric region by effective Joule heating [152]. In addition, Bae et al. [10] reported a two-terminal memristor memory based on a single VO_2_ NW at room temperature. Figure 21a shows a schematic of the memristor device with a single VO_2_ NW, voltage source and ampere meter. Figure 21b demonstrates a distinctive hysteresis loop during the MIT process under the electric field. VO_2_ stabilized in the insulating phase at a voltage less than ~0.35 V, and MIT was triggered above 0.35 V to reach the metal phase. By contrast, VO_2_ returned to the insulating phase until the voltage dropped to 0.5 V. Figure 21c displays a demonstration of a VO_2_-based two-terminal device used for information storage. When the voltage pulse is removed, the NWs maintain low resistance under bias voltage, and zero voltage bias can restore VO_2_ to its original insulating state. Interestingly, Coy et al. [149] reported the optoelectronic and all-optical multiple memory states in VO_2_. They measured resistance and near-infrared transmittance of VO_2_/SiO_2_ samples in the heating branch of the hysteresis loop at the appropriate temperature. Optical and electrical loads can be effectively combined to get several kinds of reading–writing mode. As long as the appropriate temperature was maintained, these multiple memory states were found to remain stable for at least several hours, and even indefinitely in this case. Moreover, in VO_2_-based memory devices, a mature chemical doping method or strain effect can be used to adjust the hysteresis gap of VO_2_ to get a more stable memory effect.

### 4.2. Optical Devices

#### 4.2.1. Smart Window

VO_2_ exhibits a great change in optical performance when MIT occurs. Before MIT, insulating VO_2_ (M) has high transmittance to infrared light. VO_2_ (M) changes to VO_2_ (R) and leads to low infrared transmittance when the temperature rises to the *T_c_*. This unique feature makes it attractive in the application of thermochromic smart windows. Infrared rays can pass through the glass normally at low temperatures. When the temperature is higher than a certain value, the infrared ray is isolated and the indoor temperature remains stable. Although the studies on the VO_2_-based smart window have been carried out for a long time, it has not yet reached commercial utilization. The practical applications of VO_2_ smart windows are greatly limited by their unsatisfactory intrinsic properties, e.g., relatively high critical temperature, an unpopular yellow color, poor solar energy regulation ability (∆*T*_sol_) and insufficient luminous transmittance (*T*_lum_). In the past decades, researchers have been trying to solve these problems to achieve VO_2_ thermochromic windows. Doping is a good means to control the phase transition temperature; doping with high-valence elements of tungsten (W^6+^) is one of the popular routes to reduce the phase transition with a reduction rate of ≈20–28 K per at% [153]. However, in most cases, reducing *T_c_* by doping leads to the degradation of *T*_lum_ and ∆*T*_sol_ [154]. Other solutions, including intrinsic structure optimization (e.g., particle size, crystal morphology, and porosity) [155,156] and selection of composite structure (core-shell nanostructure, hybridization, multilayer structure, etc.) [9,157,158,159], can improve VO_2_ thermochromic properties to a certain extent.

**Figure 22:**
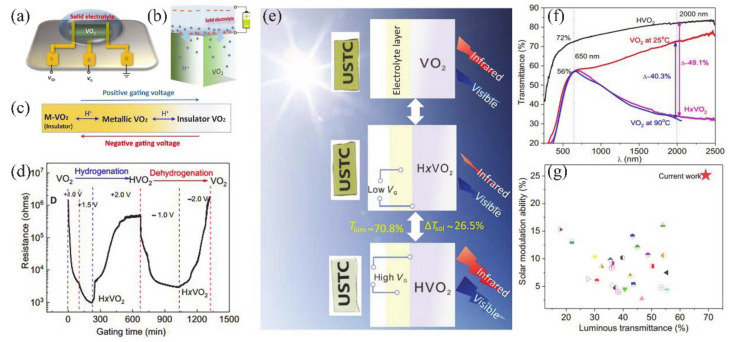
(**a**) Schematic of an electrochromic smart window device based on VO_2_ film for solid-state electrolyte gate voltage control. (**b**) Illustration of hydrogen ion movement under gating control and (**c**) reversible insulator-metal–insulator tristate phase transitions about VO_2_. (**d**) The reversible phase modulations by different voltages as a function of gating time. (**e**) Schematic diagram of electrochromic smart windows. (**f**) Optical transmittance spectra of VO_2_, H*_x_*VO_2_ and HVO_2_ thin films. (**g**) Plot of luminous transmittance *T*_lum_ versus solar modulation ability ∆*T*_sol_. The data of this work are marked with ★. Reproduced from [11], with permission from American Association for the Advancement of Science, 2019.

As we know, hydrogen is the smallest and lightest atomic element, which can effectively insert or remove the gap position of VO_2_, thereby modulating the MIT behavior [143,160]. The research results of Yoon et al. [160] demonstrated that VO_2_ stabilizes in the metal phase at a low hydrogen concentration, and the hydrogenated insulating phase (note as HVO_2_ phase) occurs again at a high hydrogen concentration. In the latest study, Chen et al. [11] took full advantage of this particular performance and developed an electrochromic smart window system based on a VO_2_ film under the solid-state electrolyte gate voltage control at room temperature. Their results broke all previous records with excellent thermochromic performances. Figure 22a–c shows the experimental schemes of gate-induced VO_2_ channel conductance modulation. The modulation mechanism is similar to the application of the IL gate FET mentioned before. This process shows that VO_2_ changes from an insulating to metallic state when a low concentration of hydrogen is injected into VO_2_; the VO_2_ appears as an insulating phase again and when the concentration of hydrogen increases continuously. Under the gate-controlled effect, the tristate transition of VO_2_ is realized. These results are in line with the work of Yoon et al. [160]. The gate-controlled VO_2_ phase transition is a reversible process. The experimental results are shown in Figure 22d. The phase transition of VO_2_ thin films can be controlled by gate voltage at room temperature, and a diagram of voltage-controlled smart window is displayed in Figure 22e. Obvious color changes are observed with three different phases of VO_2_ films. Pure VO_2_ thin films (VO_2_) and low hydrogen-doped VO_2_ thin films (H*_x_*VO_2_) have similar yellow colors, while heavily hydrogen-doped VO_2_ thin films (HVO_2_) are almost transparent. Figure 22f illustrates the optical transmittance spectra of VO_2_, H*_x_*VO_2_ and HVO_2_ thin films. The transmittance of the original VO_2_ film and metal H*_x_*VO_2_ is about 56% at 650 nm, and that of the insulating HVO_2_ film is up to 72%. In the infrared region, the transmittance difference of a pure VO_2_ film at 2000 nm is about 40.3% in the MIT process (the transmittance at 25 °C is higher than that at 90 °C), and the transmittance change from metal H*_x_*VO_2_ film to insulating HVO_2_ film is 49.1%. Figure 22g shows the comparison of the results of the work of Chen et al. [11] (★) and the previously reported data in terms of light transmittance *T*_lum_ and the solar modulation ability ∆*T*_sol_. These performances of their devices break all previous records and exceed the theoretical limit of traditional VO_2_ smart windows. This indicates that their devices are promising for energy-saving utilization.

#### 4.2.2. Photodetector

**Figure 23:**
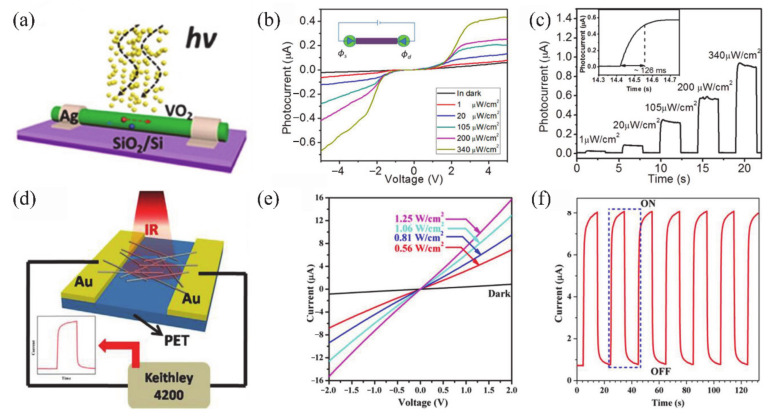
(**a**) Schematic diagram of a UV light photodetector based on a single VO_2_ microwire. (**b**) The I–V characteristic curves of VO_2_ photodetector under illumination of the different UV intensities. (**c**) Photocurrent measured under different UV intensities at a bias voltage of 4 V, and inset is the response time. Reproduced from [163], with permission from American Chemical Society, 2014. (**d**) Schematic diagram of IR light photodetector based on VO_2_ NWs. (**e**) I–V characteristics of the device at different IR radiation intensities. (**f**) Photocurrent response of the VO_2_ device under a periodic light illumination at a bias voltage of 1.5 V. Reproduced from [164], with permission from Elsevier, 2018.

Other than smart windows, the unique optical properties of VO_2_ during MIT also point to other promising applications in tunable photonic crystals [161], optical switching [162], photodetectors [79,163,164,165] and other optical devices [8,166]. Previous studies have shown that light is a sample way to trigger the MIT behavior of VO_2_. Lysenko et al. [167] reported that VO_2_ thin films can be induced to undergo reversible MIT by using a laser (energy density being 7–14 mJ cm^−2^) as an excitation source. The response of VO_2_ to light can be used to design the photodetector. Wu et al. [79] fabricated VO_2_ nanodevices based on photo-induced phase transitions of VO_2_ NWs under ultraviolet (UV) light at room temperature. Their results demonstrated that the devices exhibited excellent photoresponsive properties. The ratio of photocurrent-to-dark current was 719, and the VO_2_ NWs possess high electron mobility of 29 cm^2^V^−1^S^−1^ under 2.23 mW cm^−2^ UV irradiation. Besides, Wu et al. also developed a photodetector based on a single VO_2_ microwire with ultrahigh responsivity (R*_λ_*) and external quantum efficiency (EQE). The schematic of the device is shown in Figure 23a [163]. The photodetector showed an excellent response for ultraviolet light (λ ≈ 360–400 nm). Figure 23b shows the I–V curves under various light intensities (from 0 to 340 μW cm^−2^). Under positive and negative bias voltage, the photoelectric current of the photodetector increased with the increase of light intensity. Figure 23c reveals that the photocurrent increased to its equilibrium state immediately when the UV light was turned on. Reversely, the photocurrent fell to its initial state when the UV light was removed, and the corresponding response time was about 126 ms. The responsivity of VO_2_ photodetector was 6 and 4 orders higher than other those of graphene (or MoS_2_) and GaS, respectively [168,169,170]. This ultraviolet photodetector with a high response and EQE was the first report of nanomaterials based on a single VO_2_ microwire.

Furthermore, Li et al. reported the infrared (IR) response of self-heating VO_2_ NPs by using an Ag NW-based heater [171]. The IR photodetector is an important technology for civil and military use. As a narrow-bandgap semiconductor (0.7 eV) [163], VO_2_ will be a promising material for IR photodetectors [164,165]. Hou et al. reported an IR photodetector based on a network of as-grown VO_2_ (M1) NRs [164]. The VO_2_ (M1) NRs were synthesized by a hydrothermal method with further annealing treatment. Figure 23d illustrates the schematic of the IR photodetector device. The I–V characteristics of the device under different IR light intensity (980 nm) intensities are shown in Figure 23e. Like UV radiation, the photoelectric current of the photodetector increased with the increase of light intensity both on the positive and negative bias voltages. Figure 23f demonstrates a time-dependent photoresponse of the device. The light on and light off periods were both 10 s. Obviously, the device exhibited a “low-current” state in the dark and a “high-current” state under irradiation. The device was used with a high photosensitivity with rising and decay times being 1.6 and 1.0 s, respectively, and an excellent stability of more than 150 cycles at room temperature. Their results indicated that the networks of VO_2_ (M1) NRs were an ideal strategy for the application of IR photodetector.

### 4.3. Multi-Responsive Devices

#### 4.3.1. Strain Sensor

The lattice strain usually has significant effect on the electrical, optical and magnetic properties of materials, especially in strongly correlated electron materials. Due to the strong Coulomb force between electrons, the macroscopic properties of materials exhibit many unique physical phenomena, e.g., quantum phase transition, high-temperature superconductivity, quantum critical phenomena, giant magnetoresistance effect, metal–insulator transition, fractional quantum Hall effect, etc. VO_2_ has been proved that the MIT temperature, hysteresis loop, conductivity and domain structure are very sensitive to the strain. According to Clausius–Clapeyron formula, the relationship between MIT temperature *T_c_* of VO_2_ and strain *σ* can be expressed as follows [12],

(1) $$\frac{dT_{c}}{d\sigma }=(\varepsilon_{0}T_{c}^{0})/\Delta H$$

Among them, ΔH is the latent heat of phase transformation, $T_{c}^{0}$ is the metal–insulator phase transition temperature in the strain-free state (i.e., $T_{c}^{0}$ = 341 K) and $\varepsilon_{0}$ is the expansion of specimen along the *c*-axis during the phase transition from R phase to M phase (i.e., $\varepsilon_{0}$ ≈ 1%). Previous results have demonstrated that $dT_{c}/d\sigma$ is about 1.2 K kbar^−1^ under the uniaxial stress along *c*-axis [172]. During the phase transition process of VO_2_, the volume change of VO_2_ is much weaker than the expansion of *c*-axis. Therefore, it can be inferred from the formula that the *T_c_* of VO_2_ is more sensitive to uniaxial strain than hydrostatic pressure. This provides an effective and efficient approach to modify the MIT temperature and domain structure of VO_2_ and implies promising applications of VO_2_ in the strain sensor.

**Figure 24:**
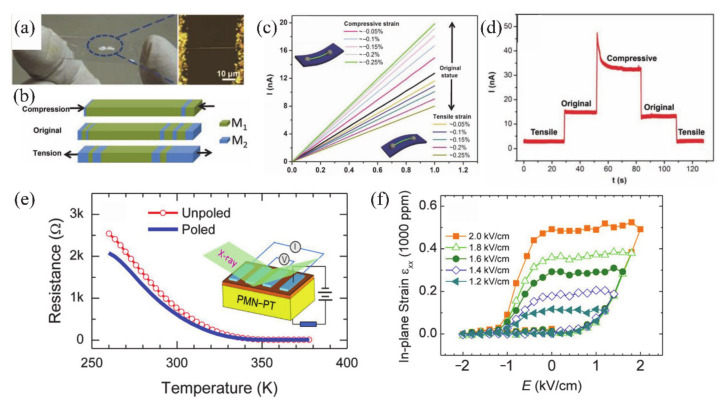
(**a**) As-fabricated strain sensor device and the corresponding optical image. (**b**) Schematic of the phase transitions of M1 and M2 with tensile and compressive strains. (**c**) The I–V curve under different tensile and compressive strains. (**d**) Fast response to the strain switch. Reproduced from [175], with permission from John Wiley and Sons, 2010. (**e**) Temperature-dependent resistance curves of VO_2_ film under the unpolarized and polarized states, and the inset shows a schematic of the VO_2_/PMNPT (111) structure. (**f**) In-plane strain for the VO_2_/PMNPT heterostructure under different electric fields. Reproduced from [144], with permission from American Chemical Society, 2014.

In the strain engineering of VO_2_, the two main ways for applying uniaxial strain are by lattice mismatch and by applying external axial strain [12,173]. Muraoka and Hiroi reported that the higher mismatch between VO_2_ and substrate should induce the higher MIT temperature [173]. Previous reports also showed that the MIT temperature of VO_2_ increases or decreases when applying the uniaxial tensile or compressive stresses along *c*-axis [173]. Moreover, the strain induced by the lattice mismatch also has a significant effect on the domain structure of VO_2_, especially for the 1D VO_2_ NWs. Generally, freestanding VO_2_ NWs exhibit single domain structure due to the absence of strain effect, while multiple domain structures will appear under the mismatch strain or external strain [12,82,174,175,176]. Hu et al. [175] fabricated a VO_2_-based strain sensor using the flexible polystyrene (PS) as substrate and studied the external strain effect on the phase transition between M1 and M2 of VO_2_ nanobeam. Figure 24a shows the fabricated strain sensor and corresponding enlarged image of the bonded nanobeam. Figure 24b illustrates the schematic of M1 and M2 domain configurations under tensile and compressive strains, and the corresponding I–V curves are displayed in Figure 24c. The increase of compressive strain leads to an increase current of VO_2_, and the increase of tensile strain reduces the current. The results indicate that M1 phase is preferred to appear and more stable under the compressive strain, whereas M2 phase tends to appear under the tensile strain. Furthermore, the response time of the strain sensor device was studied with the bias of 1 V, as shown in Figure 24d. The device exhibits a quick response to the strain switches. The results indicate that this device is suitable to serve as a strain sensor within a small mechanical strain range (±0.25%). Zhi et al. [144] fabricated VO_2_/PMN-PT (111) heterostructures and investigated the non-volatile switching modulation by ferroelectric polarization strain. The polarization strain of ferroelectric PMN-PT layer was induced by the electric field, leading to an effective resistance switching of VO_2_ between low and high resistance states. Figure 24e demonstrates the temperature-dependent resistance curves of VO_2_ film under the two polarization states (unpolarized high resistance state and polarized low resistance state). Based on the different polarization states of PMN-PT, multiple resistance states can be achieved by applying electric fields across the heterostructures appropriately (Figure 24f). More importantly, the resistance can keep unchanged after withdrawing the electric fields because of the non-volatility of ferroelectric PMN-PT layer. This provides another strategy for the application of VO_2_-based strain sensor. In addition, Chen et al. [177] mapped the pressure-temperature phase diagram of VO_2_ nanobeams under different hydrostatic pressure. Their research showed that pressure led to different phase transition temperatures of VO_2_. By combining the experiment data of optical reflectance, Raman, and electrical transport performance, they obtained the pressure-temperature phase diagram of VO_2_. The phases include M1 (I), M1′ (I), R (M), O (M) and X (M), where I and M represent insulating and metallic phases of VO_2_, respectively. Under the low temperature (<60 °C) and low pressure (15–20 GPa) conditions, VO_2_ is stabilized in M1 phase. Meanwhile, VO_2_ can maintain the metallic phase under high pressure (about 40 GPa). Their results indicate that VO_2_ can be used to develop an excellent pressure sensor.

#### 4.3.2. Gas Sensor

**Figure 25:**
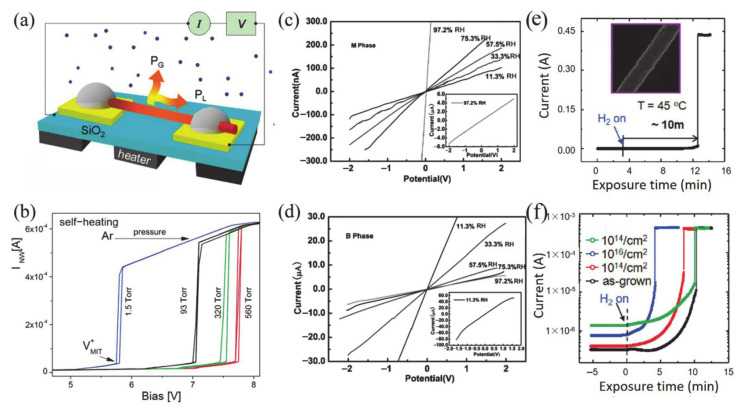
(**a**) The design and principle of operation of a VO_2_ NW gas sensor. (**b**) I–V curve of a VO_2_ NW at different Ar pressures. Reproduced from [13], with permission from American Chemical Society, 2009. (**c**,**d**) VO_2_ (M) and VO_2_ (B) nanoflowers measured in different static atmospheres, respectively. Reproduced from [178], with permission from John Wiley and Sons, 2011. (**e**) Curve of current change and hydrogen exposure time of Pd-decorated VO_2_ NW at bias voltage of 10 V. (**f**) Curve of current change with the e-beam irradiation dose and energy (0.5 MeV, red and blue curves; 0.7 MeV, green curve). Reproduced from [179], with permission from American Chemical Society, 2012.

A transition edge sensor (TES) composed of phase change materials is an effective method to detect gas atoms under ambient conditions. Strelcov et al. [13] fabricated a VO_2_ NW-based Ar gas sensor model (Figure 25a). By changing the temperature of NWs near the edge of phase transition, the conductance of NWs becomes very sensitive to small changes of the surrounding gas environment, e.g., the molecules, pressure, temperature and humidity. Therefore, VO_2_ exhibits different phase transition behaviors in such ambient atmospheres. They found that the increase of Ar pressure led to the decrease of heat dissipation of VO_2_ NW, and resulted in the increase of phase transition voltage, as shown in Figure 25b. Their results open up new opportunities for VO_2_-based gas sensors. Sensors for different kinds of gases have been developed, e.g., steam (humidity sensing) [178], hydrogen [179,180], carbon monoxide and carbon dioxide [180]. Yin et al. [178] fabricated two humidity sensors based on VO_2_ (B) and VO_2_ (M) nanoflowers with large surface-to-volume ratios and sensitive humidity sensing performances. The I–V curve of VO_2_ (M) nanoflower film-based resistive-type sensors under different static air relative humidity of 11.3–97.2% at room temperature is shown in Figure 25c. With the increase of relative humidity, the current of VO_2_ (M) slowly rises. The current changes up to about an order of magnitude when the relative humidity reaches 97.2%. In contrast, the conductivity of the VO_2_ (B) nanoflower film changed conversely with the increase of relative humidity (Figure 25d). The resistance of VO_2_ returned to its original state when the sensor was out of the humid environment. Most importantly, the VO_2_ (M) sensor is more sensitive in a high relative humidity, whereas the VO_2_ (B) sensor is more sensitive in low relative humidity. Therefore, the VO_2_ nanoflowers with large surface-to-volume ratios are good candidates for developing humidity sensors.

On the other hand, a hydrogen sensor is an important research topic. Byon et al. [179] developed a highly responsive and selective hydrogen sensor based on MIT of Pd-decorated VO_2_ NWs. The conductivity of VO_2_ has a significant four-times increase when under the irradiation of an electron beam, while the temperature of MIT varies moderately. Figure 25e shows the current in the as-grown Pd-decorated VO_2_ NW as a function of hydrogen exposure time under a bias voltage of 10 V and a temperature of 45 °C. The current evolution can be divided into two stages. In stage I, the current of VO_2_ NW increases slowly with the exposure time of hydrogen. This slow process takes place in a few minutes and depends on electrical conductivity and ambient temperature. In stage II, the current of NW increases about 1000-fold after exposing in hydrogen about 10 min. This ultra-fast process takes several nanoseconds or microseconds to reach the metal phase. The slow process is considered as a complex process, which may involve the formation of hydrogen atoms, the interaction with Pt, the migration of hydrogen atoms to the surfaces of oxides and the infiltration into the volume region of oxides, etc. In the second ultra-fast process, the hydrogen matrix acts as an electron donor and increases the carrier density in the conduction band, so that the conductivity of VO_2_ has a significant increase. Figure 25f shows the evolution of current with exposure time of Pd-modified VO_2_ NWs under different irradiation conditions (i.e., energy and dose). It is obvious that VO_2_ undergoes different MIT behaviors with the change of electron beam irradiation conditions when it is exposed to hydrogen. Obviously, the response time is significantly reduced to less than 4 min when it increases the dose up to 10^16^/cm^2^, and only half of the time without irradiation (~10 min). The increase of conductivity induced by irradiation accelerates the self-heating effect and leads to the decrease of response time, working temperature and voltage. Moreover, the sensor also shows the ability to selectively detect hydrogen in gases (oxygen, carbon monoxide and ethylene).

#### 4.3.3. Thermal and Laser Sensors

**Figure 26:**
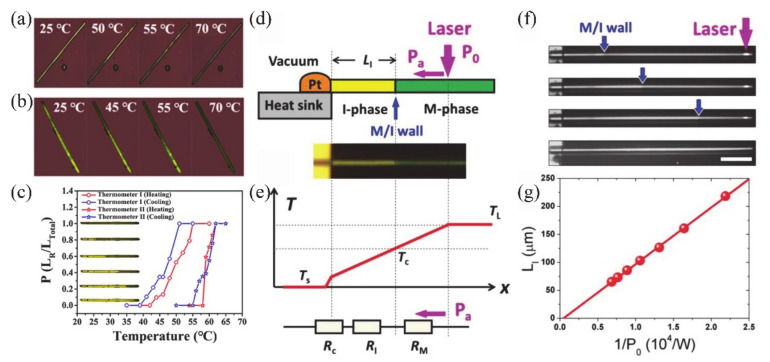
(**a**,**b**) Optical images of VO_2_ NWs during the heating process. (**c**) Plots of domain wall position along the graded-doping VO_2_ (M) NWs under the different temperatures and the corresponding optical images of an annealed thermometer with the increasing temperature. Reproduced from [181], with permission from American Chemical Society, 2017. (**d**) Schematic of the working principle about a near-field power meter based on the cantilevered VO_2_ beam, and the optical image shows the position of the domain wall along the beam. (**e**) Schematic of temperature profile along the VO_2_ microbeam. (**f**) Optical images of the domain wall movement under increasing (bottom to top) laser power. (**g**) Plot of the length of the insulating domain and the reciprocal of incident laser power. Reproduced from [182], with permission from John Wiley and Sons, 2015.

With the rapid development of nanotechnology, it is urgent to develop more accurate and quantitative measurement technology on nanostructures. So far, powerful solutions for in situ and local temperature measurements of micro-nanoobjects remain limited. We know that VO_2_ is very sensitive to temperature, and MIT behavior occurs under certain temperature conditions. VO_2_ nanostructures (NWs, NSs) usually exhibit a single M domain structure under optical microscopy without external stimuli. When they are exposed to external stimuli, such as strain and heat, VO_2_ NWs exhibit distinct M–R domains until they are completely converted to R domains [12,82,176]. Therefore, aside from the strain sensing mentioned above, thermal sensing is also a typical application of VO_2_. Shi et al. [181] took advantage of the properties of thermal sensing and developed optically readable thermometers based on graded H-doping NWs. Figure 26a,b shows the evolution of M–R domains during heating process. It is obvious that M domain decreases and R domain increases with the raising of temperature. Moreover, the length of the metal domain of NWs rises linearly with the increase of ambient temperature. (Figure 26c). It is noteworthy that the NW-based thermometer can move in the heating and cooling processes outside the hysteresis-free domain; thus, sensitive real-time temperature monitoring is realized, and an excellent performance via good relative sensitivity (17.4%/K) and temperature resolution (0.026 K) is achieved. However, there are still some challenges in its practical application. For instance, the modulation range of MIT temperature is limited. Therefore, more research is needed to develop VO_2_-based thermal sensors.

Another interesting application based on VO_2_ NWs is about the effect of laser on the M–R domain wall of VO_2_ movement. Laser heating can also induce the MIT behavior of VO_2_ [183]. This property can be exploited to develop a novel VO_2_-based laser power meter. Cheng et al. [182] demonstrated a multifunctional power meter based on VO_2_ NWs, and the light absorption and heat transfer directly can be quantified in the near field length range (Figure 26d–g). The laser struck on the one end of the NW, and the laser power flowed unidirectionally toward the substrate. Once enough power is obtained, the metal domain appears, and the temperature of the domain wall just turns into 68 °C. In fact, the length of the insulating domain (L_I_) decreases with the increasing of the laser power (P_0_), which corresponds to the domain distribution of VO_2_ NW at different temperatures. By changing the optical power (laser or electron beam), the optical absorptivity of VO_2_ NWs can be determined from the slope of L_I_ − 1/P_0_. These near field power meter can be used for the quantitative study of thermal and optical fields in nanoscale.

### 4.4. Other Devices

**Figure 27:**
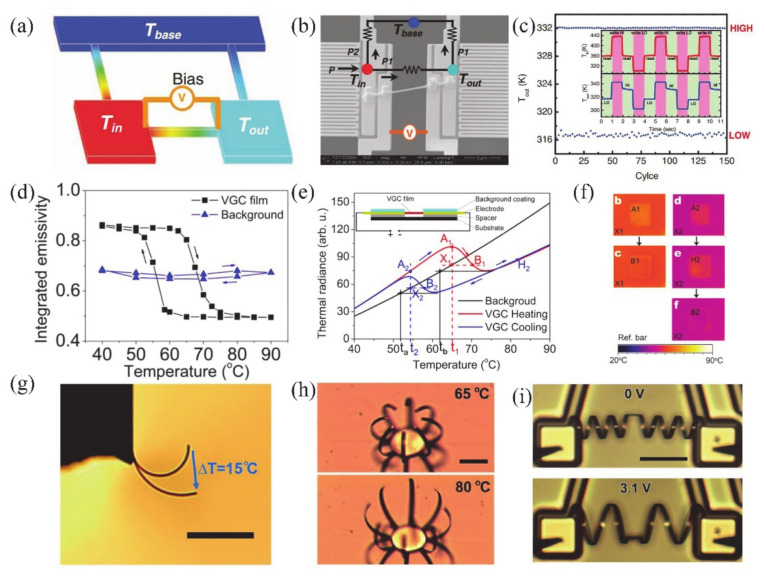
(**a**) A schematic illustration of a thermal memory device. (**b**) SEM image of a thermal memory device. (**c**) High/low temperature status over 150 cycles by using a 1 s heating pulse and 1 s cooling pulse; the inset shows the process of write–high-read–write–low-read over three cycles. Reproduced from [184], with permission from John Wiley and Sons, 2011. (**d**) Temperature emissivity of VGC film on black tape and background coating. (**e**) Thermal radiance from the background (black line) coating and free-standing VGC film, (red line refers to heating process, blue line refers to cooling process), and the inset shows the structure of VGC-based device; and (**f**) the thermal images of the direct and delayed heating camouflage processes are shown on the right. Reproduced from [186], with permission from American Chemical Society, 2015. (**g**) Optical image of VO_2_ cantilever change with a temperature change of 15 °C; the scale bar is 50 μm. (**h**) Optical image of VO_2_ microactuator with a “palm” structure under temperatures of 65 °C and 80 °C. Reproduced from [188], American Chemical Society, 2012. (**i**) The geometry of VO_2_ double coil under at 0 V and 3.1 V; the scale bar is 100μm. Reproduced from [189], with permission from John Wiley and Sons. 2014.

In addition to the above well-known applications, the MIT of VO_2_ is also related to other aspects. We mentioned earlier that the voltage can drive the MIT of VO_2_ with an appearance of hysteresis, which implies a potential application in electrical memory devices. It is interesting to note that the hysteresis caused by thermally driven phase transitions can also be used to store thermal information. Xie et al. [184] demonstrated a thermal memory device based on VO_2_ nanobeams. This device can be used for storing and retaining thermal information, and the temperature states are used as input and output. The thermal memory device includes an input (*T*_in_), an output (*T*_out_) and one heat conduction channel that bridges the two terminals together. Two terminals are connected and then connected to a substrate (*T*_base_), as shown in Figure 27a,b. VO_2_ nanobeam was used as a controllable thermal channel to realize the response between *T*_in_ and *T*_out_, as shown in Figure 27c. The results prove that the characteristics of thermal memory can be modulated by changing the voltage. To determine the switching performance and repeatability of thermal memory, they used heating and cooling pulses to perform repeated high-read–write–low-read cycles. Repeated cycles of more than 150 times shows that both high and low states were reliable, reproducible and non-degenerative.

The excellent thermal emissivity and large negative differential thermal emissivity of VO_2_ make it a candidate material for infrared camouflage and thermal regulation [185]. Xiao et al. [186] fabricated VO_2_/graphene/carbon nanotube (VGC) flexible films with an adjustable emissivity, as shown in Figure 27d. The thermal emissivity of the VGC films is ~0.86 at 40 °C and ~0.49 at 90 °C. The large adjustability of emissivity indicates that the VGC film is a promising active thermal camouflage material. Near the *T_c_*, the thermal radiation of VGC thin films is greatly reduced during the heating process; on the contrary, it increases when cooling. Although the temperature of VGC film is slightly higher than that of background (Figure 27e), its total thermal radiation is almost constant. Therefore, infrared cameras cannot distinguish them, and the process can be easily controlled by electronic adjustment. This infrared camouflage can be used at room temperature by doping to reduce the *T_c_* of VO_2_.

In addition to reversible changes in resistivity and light transmittance, the reversible strain will also occur during the process of MIT. When VO_2_ lattice changes from monoclinic (insulating) to rutile (metal) structure, VO_2_ shrinks under a strain of *ε* ≈ 1% along the *c*-axis of the rutile phase and expands in other two directions [12]. Such an abrupt strain of 1% makes VO_2_ a good active layer for an actuator. Rúa et al. [187] developed a thermally activated actuator based on cantilever bending of polycrystalline VO_2_ coating. The VO_2_ thin film was deposited on a silicon microcantilever. The fabricated bimorph cantilever exhibited thermally-driven bending during MIT. The curvature change and amplitude were ~2000 m^−1^ and ~115 μm, respectively. This is much better than the traditional bimorph actuator based on differential thermal expansion. Besides, in recent years, a series of Cr/VO_2_ thin film bimorph drivers with different patterns and structures have been reported [188,189,190,191]. Liu et al. developed micro-fabrication steps to design various shapes and structures of micro-Cr/VO_2_ thin film-based actuators (Figure 27g–i) [188,189]. These actuators can be triggered by different types of stimuli, e.g., heating, electric field, strain and light. The actuators had a high bending amplitude and the actuation frequency was up to ~6 kHz [188]. Besides, Cr/VO_2_ thin film actuators were further made into coils, which can provide up to 200,000 rpm of torsional motion. The peak power density reached 39 kW/kg, and the measurement of one million driving cycles indicated that the material has strong fatigue resistance. This miniature bending and twisting machine may become a key component of robots, artificial muscles, intelligent shutters and biochemical drug delivery. Ma et al. [190] deposited VO_2_ on carbon nanotubes (CNT) and realized flexible actuators. By using W-doping technology to reduce the *T_c_* of VO_2_, these VO_2_/CNT flexible actuators can work under the stimuli of sunlight or human body temperature.

### 4.5. Summary

In this section, the properties and related applications of VO_2_ were introduced. Almost all the application prospects are based on the phase transition characteristic of VO_2_. The electrical changes caused by MIT make VO_2_ promising in applications such as electronic switches, FET and memory devices. Due to the change of optical properties caused by MIT and the electrical properties of VO_2_ being sensitive to light, VO_2_ can be used to develop smart windows, photodetectors, etc. The MIT of VO_2_ is sensitive to strain, temperature and surrounding gas environment, which makes VO_2_ as a candidate material in multiple types of response sensors. The strain produced by the MIT of VO_2_ makes it useful for micro or nanoactuators. Moreover, VO_2_ has great potential for developing infrared camouflage and thermal regulation.

## 5. Summary and Outlook

In this review, we first introduced several phases of VO_2_, including monoclinic VO_2_ (M), tetragonal VO_2_ (R) and metastable VO_2_ (A), VO_2_ (B) and VO_2_ (C); and new phases of VO_2_ (D) and VO_2_ (P). Under certain conditions, these phases can be transformed into each other. Among them, both VO_2_ (A) and VO_2_ (M) exhibit typical MIT behavior, and VO_2_ (M) is widely focused on because its *T_c_* is close to room temperature. Then the preparation strategies of VO_2_ nanostructures are discussed in the second section. The fabrication of the thin films and various LDSs (e.g., NPs, NWs, NRs, NSs, NBs, NDs, nanorings, microspheres and micro- or nano-plates) of VO_2_ via hydrothermal method, CVD, PLD, sol–gel method, magnetron sputtering, electrospinning and MBE was comprehensively summarized. The hydrothermal method and CVD are mainly used to prepare LDSs of VO_2_. Such chemical synthesis processes easily produce by-products, such as metastable phases or non-stoichiometric amounts of VO_2_. Therefore, it is very important to strictly control the synthesis conditions. PLD, magnetron sputtering, MBE and sol–gel are commonly used to grow VO_2_ thin films. Interestingly, by precisely controlling the deposition conditions, PLD also can fabricate various LDSs. Finally, we discussed the performance and corresponding device applications of VO_2_, including electrical and optical devices, various sensor devices (e.g., strain sensor, gas sensor, thermal sensor and laser sensor) and other applications (e.g., thermal memory, thermal camouflage and actuators). These show that VO_2_ has great potential applications in advanced multifunctional devices.

As mentioned above, a series of remarkable breakthroughs in the fabrication of VO_2_ have been achieved, and many advanced applications have been widely studied. However, there are still some challenges that should be addressed, some of which follow:

(1) Regarding the fabrication aspect, it is still a challenge to precisely control the sizes and alignments of VO_2_ products. The size of NWs can directly affect the MIT behavior of VO_2_; thus, it is important to accurately control the size of NWs. For the hydrothermal method, it is also a tough task to fabricate ultra-small size (<20 nm) VO_2_ nanostructures. As we mentioned earlier, the decomposition rate of the precursor (*r*_d_) and the growth rate of the grain (*r*_g_) are two important factors determining the particle size of VO_2_. Therefore, in the hydrothermal method, VO_2_ nanostructures with ideal size can be synthesized by controlling *r*_d_ and *r*_g_. Besides, due to the polymorphs of VO_2_ and the complicated growth process, it is still a challenge to synthesize the VO_2_ (M) in one step via hydrothermal method. Therefore, the one-step synthesis of VO_2_ (M) needs to be further explored, and the choice of surfactant or catalyst and the precise control of the synthesis process can be considered in the process of hydrothermal synthesis. For the CVD method, it is a challenge to control the orderly alignment of VO_2_ NWs. As we mentioned before, one of the strategies to control the growth orientation of VO_2_ NW is to form a template by modifying the surface structures of the substrate or depositing a patterned seed layer. Meanwhile, catalysts also may be necessary to promote the growth of aligned VO_2_ NWs. On the other hand, for the PLD, magnetron sputtering and MBE, proper substrate and deposition conditions are key factors for the fabrication of VO_2_ thin films of high-quality. Besides, surface pre-treatment always is needed to improve the surface status of the substrate and thereby decrease the defects in as-grown thin films.
(2) For the application aspect, almost all the application prospects are based on the phase transition characteristic of VO_2_. Therefore, controlling the MIT temperature of VO_2_ is very important in the device applications. However, the phase transition temperature of VO_2_ is about 68 °C, which is still too high for applications in electronic devices. In particular, for the VO_2_-based switch devices, smart windows, laser power meters, thermally activated actuators, etc., the relatively high *T_c_* results in high energy consumption and damage to the device. At present, the conventional approach to control the *T_c_* is doping, and the *T_c_* can be reduced to near room temperature. However, the corresponding slow transition rate and large hysteresis window during the MIT will limit the high-speed responses of VO_2_-based devices. Therefore, to find an efficient way to tune *T_c_* without affecting the transition rate and hysteresis window of MIT is very necessary to improve the performances of VO_2_-based devices. On the other hand, due to the intrinsic properties of VO_2_, e.g., the low resistance of insulating phase in doped VO_2_, the low visible light transmittance of VO_2_ smart windows, etc., there is a big gap to realize the practical applications of VO_2_-based devices. Therefore, in order to improve the performance of VO_2_-based devices, a good strategy is to combine VO_2_ with other functional materials (ferroelectric multifunctional layer, etc.) to form composite structures.
(3) In recent years, phase separation and coexistence (i.e., metal–insulator domain structures) of VO_2_ became a hot topic due to its importance in the deep understanding of the mechanism of phase transition and the controllability of phase transition behavior. More recently, we found that a macroscopic defect, i.e., a void, has significant influence over the metal–insulator domain structures and their evolutionary paths during the phase transition. By analyzing the distribution of stress field near the defect and the evolution paths of domain structures, we suppose that the defect-induced local stress should play an important role. Defect-induced local stress concentration leads to the pinning of the phases and high agreement in the evolutionary paths of domain structures during the phase transition. Moreover, the shape, position and number of macroscopic defects, which are determinants of the distribution of the stress fields in the VO_2_ nanostructures, could be precisely controlled by employing a focused ion beam (FIB) and nanoindentation. This results in the MIT temperature, hysteresis loop, conductivity and domain structure of VO_2_ nanostructures also being precisely controlled. This provides a novel strategy to precisely control the phase transition temperature and behaviors.

Nowadays, with the development of fabrication and micromachining technologies, the above problems are attracting much attention and are gradually being taken on. The improved MIT and multi-functional properties will promote the practical applications of VO_2_-based functional devices. Besides, numerous new application concepts based on VO_2_ also have been proposed—e.g., an ultrafast response flexible breath sensor [192], artificial skin [193] and artificial synapses [194]. The emergence of new concepts is expected to expand the applications of VO_2_ and to acquire more opportunities.

## Figures and Tables

**Table 1:** Common synthetic environment, crystallography data and corresponding comments of VO_2_ polymorphs.

Polymorphs		Space Group	Unit Cell Parameters				Common Reaction Conditions	Comment	Reference
			a	b	c	αβγ			
VO_2_ (M)	VO_2_ (M1)	*P* 2_1_/c	5.715	4.554	5.385	β = 122.6°	V source: V_2_O_5_, VH_4_VO_3_ Reductant: H_2_C_2_O_4_, N_2_H_4_ Surfacant: polyvinylpyrrolidone (PVP), polyethylene glycol (PEG) Temperature: ~150–260 °C Time: a few hours to a few days	Most of the research and applications are based on the MIT of VO_2_ (M).	[21]
	VO_2_ (M2)	*C* 2/*m*	9.067	5.797	4.526	β = 91.88°			
VO_2_ (R)		*P* 4_2_/*mnm*	4.554	4.554	2.85	α = β = γ = 90°		The high-temperature rutile phase of VO_2_.	[22]
VO_2_ (A)		*P* 4_2_/*nmc*	8.434	8.434	7.678	α = β = γ = 90°		Another phase with MIT Behaviour with *T_c_* = 435 K	[27,29]
VO_2_ (B)		*C* 2/*m*	12.03	3.693	6.42	β = 106.6°		It has layer structure, which suitable for electrode materials and thermal sensitive materials of batteries.	[24,30]
VO_2_ (C)		*I* 4/*mmm*	3.7211	15.421	N/A	N/A		The structure of VO_2_ (C) consists of VO_5_ square pyramids, each of which shares its four base edges with four adjacent VO_5_ square pyramids.	[28]
VO_2_ (D)		*P* 2/*c*	4.597	5.684	4.913	β = 89.39°		VO_2_ (D) exhibits magnetic properties, and it can be transformed to VO_2_ (M) at 300 °C.	[25]
VO_2_ (P)		*Pbnm*	4.890	9.390	2.930	β = 90°		VO_2_ (P) was synthesized by simple chemical reaction by Wu et al. and it can be transformed to VO_2_ (M) by rapid annealing.	[26]

**Table 2:** The strategies for the growth of VO_2_.

Method	Structure	Phase	Advantages	Disadvantages	References
Hydrothermal method	NPs, NWs, NRs, NSs, nanorings, microsheres	VO_2_ (A), VO_2_ (B), VO_2_ (C), VO_2_ (D), VO_2_ (P), VO_2_ (M)	1. High crystallinity of products 2. Simple process 3. High yield 4. Various structures can be prepared.	1. Narrow reaction temperature 2. High pressure	[33,49,50,54,58,64,67]
CVD	Thin film, NWs, microplates	VO_2_ (M), VO_2_ (B)	1. Simple equipment 2. High flexibility	1. Low deposition rate 2. Difficult to control the size of NWs	[75,76,81,82]
PLD	Thin film, NDs, NWs, NBs, NRs, nanoplatelets	VO_2_ (M)	1. Simple process 2. Low temperature 3. High deposition rate 4. In situ growth	1. Low yield 2. Small film area 3. Easy to from particle	[16,88]
Sol–gel method	Thin film, nanopowders, ND array	VO_2_ (M)	1. Material composition can be strictly controlled 2. Easy to dope 3. Simple equipment	1. Low film-forming quality 2. Long reaction time	[104,106,107]
Magnetron sputtering	Thin film	VO_2_ (M)	1. High film formation rate 2. Good crystallinity 3. Low substrate temperature	1. Poor consistency between film composition and target material 2. Poor process stability	[114,115]
Electrospinning	Nanofibers	VO_2_ (M)	1. Controllable diameter of nanowires 2. Simple equipment and low cost	1. Low fiber strength 2. Low yield 3. The products are greatly affected by ambient temperature and humidity. 4. Difficulty in separating fibers	[42]
MBE	Thin film	VO_2_ (M)	1. Clean growth environment 2. Low growth temperature 3. Good crystal integrity 4. Easy to dope	1. Expensive equipment and high maintenance costs 2. High vacuum requirements	[116,117,118]

## Data Availability

No new data were created or analyzed in this study. Data sharing is not applicable to this article.
